# High-throughput screening and genome-wide analyses of 44 anticancer drugs in the 1000 Genomes cell lines reveals an association of the *NQO1* gene with the response of multiple anticancer drugs

**DOI:** 10.1371/journal.pgen.1009732

**Published:** 2021-08-26

**Authors:** Farida S. Akhtari, Adrian J. Green, George W. Small, Tammy M. Havener, John S. House, Kyle R. Roell, David M. Reif, Howard L. McLeod, Timothy Wiltshire, Alison A. Motsinger-Reif

**Affiliations:** 1 Department of Biological Sciences, North Carolina State University, Raleigh, North Carolina, United States of America; 2 Bioinformatics Research Center, North Carolina State University, Raleigh, North Carolina, United States of America; 3 Pharmacotherapy and Experimental Therapeutics, University of North Carolina at Chapel Hill, Chapel Hill, North Carolina, United States of America; 4 Biostatistics and Computational Biology Branch, National Institute of Environmental Health Sciences, Durham, North Carolina, United States of America; 5 Geriatric Oncology Consortium, Tampa, Florida, United States of America; 6 USF Taneja College of Pharmacy, Tampa, Florida, United States of America; 7 Center for Pharmacogenomics and Individualized Therapy, University of North Carolina at Chapel Hill, Chapel Hill, North Carolina, United States of America; Broad Institute, UNITED STATES

## Abstract

Cancer patients exhibit a broad range of inter-individual variability in response and toxicity to widely used anticancer drugs, and genetic variation is a major contributor to this variability. To identify new genes that influence the response of 44 FDA-approved anticancer drug treatments widely used to treat various types of cancer, we conducted high-throughput screening and genome-wide association mapping using 680 lymphoblastoid cell lines from the 1000 Genomes Project. The drug treatments considered in this study represent nine drug classes widely used in the treatment of cancer in addition to the paclitaxel + epirubicin combination therapy commonly used for breast cancer patients. Our genome-wide association study (GWAS) found several significant and suggestive associations. We prioritized consistent associations for functional follow-up using gene-expression analyses. The NAD(P)H quinone dehydrogenase 1 (*NQO1*) gene was found to be associated with the dose-response of arsenic trioxide, erlotinib, trametinib, and a combination treatment of paclitaxel + epirubicin. *NQO1* has previously been shown as a biomarker of epirubicin response, but our results reveal novel associations with these additional treatments. Baseline gene expression of *NQO1* was positively correlated with response for 43 of the 44 treatments surveyed. By interrogating the functional mechanisms of this association, the results demonstrate differences in both baseline and drug-exposed induction.

## Author summary

In the burgeoning field of personalized medicine, genetic variation is recognized as a major contributor to patients’ differential responses to drugs. Lymphoblastoid cell lines (LCLs) are a consistent and convenient representation of cells used for *in vitro* research. Human genome sequencing with LCLs can identify new genes that influence individuals’ drug responses, including the dose-response relationship, which describes the relationship between physiological response and the amount of exposure to a substance. In this work, we conduct high-throughput screening and genome-wide association mapping using 680 LCLs from the 1000 Genomes Project to identify new genes that influence individual response to 44 widely used anticancer drugs. We found the *NQO1* gene to be associated with the dose-response of several drugs, namely arsenic trioxide, erlotinib, trametinib, and the paclitaxel + epirubicin combination, and performed follow-up analyses to better understand its functional role in drug response. Our results indicate *NQO1* expression is correlated with increased drug resistance and provide some evidence that SNP rs1800566 influences drug response by altering protein activity for these four treatments. With further research, *NQO1* has potential use as a therapeutic target, for example, suppressing *NQO1* expression to increase sensitivity to particular drugs.

## Introduction

A major goal of precision medicine is improved prediction of response to treatment in cancer patients, who exhibit inter-individual variability in both drug efficacy and drug toxicity due to tumor heterogeneity and host genetic variation [1–4]. Adverse drug effects range from mild reactions such as rash or nausea to severe reactions that can include peripheral neuropathy, hematotoxicity, or febrile neutropenia [5, 6]. Genetic, epigenetic, and environmental factors, along with the interactions between these factors, combined with inherent tumor heterogeneity, contribute to drug-response variability [1–4]. As detailed in the PharmGKB database [7], there are a growing number of pharmacogenomic biomarkers. For example, head and neck cancer patients carrying the *A* allele at rs2227983 in their epidermal growth factor receptor (*EGFR*) gene have greatly increased treatment response and survival after cetuximab (an EGFR antagonist) treatment. Likewise, dosage titration of mercaptopurine, which is used to treat acute lymphoblastic leukemia, is recommended in patients with polymorphisms in the *TPMT* gene due to increased toxicity [8]. Despite these successes, predictive biomarkers remain elusive for a large number of anticancer drugs [3, 9], and further elucidation of genetic contributions to treatment efficacy and toxicity remains a critical goal of pharmacogenomics.

Lymphoblastoid cell line (LCL) experiments of anticancer drug response have demonstrated that drug response is a heritable trait, leading researchers to identify host genetic variants associated with dose-response phenotypes in this system [10–12]. As reviewed by Jack et al. [13], the LCL model offers several advantages. The limited number of confounders and large sample sizes compared to pharmacogenomics studies in clinical trials and the ready public availability of genotyping data make the LCL model a cost-effective and efficient system for discovering new genetic associations. Previous large-scale studies on *in vitro* drug response have focused on cancer cell lines to identify associations between somatic mutations and drug response [14, 15]. While it has long been established that somatic mutations in a tumor affect drug efficacy, studies have shown that the contribution of germline variants can be as great or greater than that of somatic mutations [9, 16, 17]. Hence, in cancer pharmacogenomics, both somatic and germline variations must be identified to maximize drug efficacy and minimize drug toxicity. LCLs, which are typically generated from peripheral blood mononuclear cells, represent the germline variation in the host genome and thus allow for interrogation of the effects of germline variants on drug response. While there are limitations to the model (e.g., minimal expression of drug transporter enzymes), it has been successfully used in a range of pharmacogenomics studies [17–21]. The numerous successes in using the LCL model to identify clinically relevant pharmacological phenotype-genotype associations [17–21] include the association of single nucleotide polymorphisms (SNPs) in the *MGMT* gene with temozolomide response [22], the identification of genetic variants associated with cytotoxicity to platinating agents across populations [23], and the association of *FKBP5* expression levels with cytarabine cytotoxicity [24, 25].

In the current study, we present the results of the largest LCL screen of anticancer drugs to date. We assayed 680 cell lines from the 1000 Genomes Project across 44 anticancer drugs at six doses of each drug, resulting in 179,520 drug-dose-cell line combinations. The LCLs from the 1000 Genomes Project represent true global ethnic and racial diversity, making the results of our study applicable to multiple global populations. The drug treatments considered in this study represent nine drug classes widely used in the treatment of cancer in addition to the paclitaxel + epirubicin combination therapy commonly used for breast cancer patients [26–28]. Using genome-wide association studies (GWAS) to find SNPs associated with the cytotoxicity of 44 anticancer drug treatments, we report several genome-wide significant (p < 10^−8^) and suggestive (p < 10^−6^) associations. We prioritize consistent associations for functional follow-up using gene-expression analyses. Further, our functional work interrogates the potential mechanism of action of our findings.

## Results

### GWAS results

Drug treatment responses in each of the 680 cell lines were assessed with the alamarBlue assay across six doses of each of the 44 drug treatments. Pilot experiments were conducted to determine treatment-specific dose ranges to maximize the variability in dose-response across individuals. Table 1 shows treatments and their drug classes, and Table A in S1 Text shows the six concentrations used for each treatment. We used a carefully developed quality control pipeline to process dose-response assays, the details of which are described in Brown et al. [22, 29]. We downloaded genetic data for each cell line from the 1000 Genomes database [30] (https://www.internationalgenome.org/data-portal/data-collection/phase-1) and, after routine quality control, used 1,510,701 SNPs for GWAS. Quality control details can be found in Abdo et al. [31]. To avoid the assumptions and noise of curve fitting, we ran a multivariate analysis of covariance (MANCOVA) for each SNP across the genome using previously developed multivariate analysis of covariance genome-wide analysis (MAGWAS) software [32]. As described in detail in Brown et al. [32], we used the viability at each dose point as a vector response in the MANCOVA model tested principal components, other covariates, and a genotypic-encoded SNP variable for association. We used this MANCOVA model to test each SNP across the genome and corrected for multiple comparisons at the significant and suggestive levels based on the effective number of variants across the genome [33].

**Table 1:** Anticancer drug treatments used for the drug-response assays by drug class.

	Drug treatment	Drug class
1	Hydroxyurea	Anti-metabolite
2	MitomycinC	DNA alkylating agent
3	Temozolomide	
4	Paclitaxel + Epirubicin	Combination treatment
5	Etoposide	Epipodophyllotoxins
6	Teniposide	
7	Daunorubicin	Anthra-cyclines/cendiones (Topoisomerase II inhibitors)
8	Doxorubicin	
9	Epirubicin	
10	Idarubicin	
11	Mitoxantrone	
12	Topotecan	
13	Docetaxel	Microtubule binding agents
14	Paclitaxel	
15	Vinblastine	
16	Vincristine sulfate	
17	Vinorelbine	
18	Azacytidine	Nucleosides
19	Cladaribine	
20	Cytosine beta-D-arabinoside	
21	Fludarabine	
22	Gemcitabine	
23	5-Fluorouracil	Fluoropyrimidines
24	Fluoro-deoxyuridine	
25	Arsenic trioxide	Other
26	Carboplatin	Platinum agents
27	Oxaliplatin	
28	Apatinib	Tyrosine kinase inhibitors
29	Axitinib	
30	Cabozantinib	
31	Crizotinib	
32	Dasatinib	
33	Dovitinib	
34	Erlotinib	
35	Ibrutinib	
36	Masatinib	
37	Nilotinib	
38	Nintedanib	
39	Sorafenib	
40	Sunitinib	
41	Tivantinib	
42	Trametinib	
43	Vandetanib	
44	Vemurafenib	

The 44 anticancer drug treatments, listed by drug class, used for the drug-response assays in LCLs in this study.

Table 2 reports suggestive genome-wide significant associations (p < 10^−6^) that comprise 40 unique SNPs across 21 anticancer treatments. Further, we report 10 SNP-drug associations at a genome-wide significance level of p < 10^−8^. We used the Ensembl Variant Effect Predictor (VEP) [34] to classify the suggestively significant SNPs. The two largest categories are intron variants, with 53% of the SNPs, and intergenic variants, with 20% of the SNPs. The SNPs in these results are annotated across 18 unique genes.

**Table 2:** SNPs significantly associated with drug response.

	Drug	Chr	RSID	-log10 (pvalue)	Most severe consequence	Host gene symbol	Host gene Ensembl ID
1	Cladaribine	7	rs540157	6.52	regulatory_region_variant	*-*	-
2	Cladaribine	9	rs72706422	6.14	intergenic_variant	*-*	-
3	Dovitinib	11	rs7480726	6.16	upstream_gene_variant	*-*	-
4	Dovitinib	11	rs7930221	6.81	upstream_gene_variant	*-*	-
5	Epirubicin	10	rs1125411	7.05	intergenic_variant	*-*	-
6	Epirubicin	10	rs7911302	6.97	intergenic_variant	*-*	-
7	Gemcitabine	12	rs11043377	6.15	intergenic_variant	*-*	-
8	Gemcitabine	12	rs6486806	7.41	intergenic_variant	*-*	-
9	Hydroxyurea	2	rs13420950	6.09	intergenic_variant	*-*	-
10	Oxaliplatin	10	rs10826348	7.71	intergenic_variant	*-*	-
11	Oxaliplatin	10	rs1112962	6.24	intergenic_variant	*-*	-
12	Paclitaxel	2	rs1107718	7.95	intergenic_variant	*-*	-
13	Paclitaxel	9	rs4740816	6.23	upstream_gene_variant	*-*	-
14	Tivantinib	18	rs11662580	6.52	intergenic_variant	*-*	-
15	Hydroxyurea	8	rs13261597	6.01	intron_variant	*ADRA1A*	ENSG00000120907
16	Vandetanib	7	rs10273337	6.04	intron_variant	*AGAP3*	ENSG00000133612
17	Vemurafenib	2	rs4664521	6.31	intron_variant	*CACNB4*	ENSG00000182389
18	Vemurafenib	2	rs9784082	6.58	intron_variant	*CACNB4*	ENSG00000182389
19	Nintedanib	7	rs798933	6.10	intron_variant	*CPED1*	ENSG00000106034
20	Gemcitabine	7	rs216706	6.29	intron_variant	*CREB5*	ENSG00000146592
21	Vinorelbine	8	rs1478275	6.04	intron_variant	*CSMD1*	ENSG00000183117
22	Vemurafenib	6	rs12191002	6.07	intron_variant	*GMDS-DT*	ENSG00000250903
23	Gemcitabine	10	rs17142881	7.10	intron_variant	*ITIH5*	ENSG00000123243
24	Vinblastine	16	rs1693956	6.12	intron_variant	*LINC01081*	ENSG00000268754
25	Oxaliplatin	10	rs11006706	8.91	intron_variant	*MKX-AS1*	ENSG00000230500
26	Carboplatin	20	rs6010746	6.27	intron_variant	*MRGBP*	ENSG00000101189
27	Arsenic trioxide	16	rs11641233	6.05	3_prime_UTR_variant	*NFAT5*	ENSG00000102908
28	Erlotinib	16	rs11639947	8.39	intron_variant	*NFAT5*	ENSG00000102908
29	Erlotinib	16	rs11641233	8.16	3_prime_UTR_variant	*NFAT5*	ENSG00000102908
30	Erlotinib	16	rs12232410	9.10	3_prime_UTR_variant	*NFAT5*	ENSG00000102908
31	Erlotinib	16	rs12447326	9.17	intron_variant	*NFAT5*	ENSG00000102908
32	Erlotinib	16	rs2361838	7.91	intron_variant	*NFAT5*	ENSG00000102908
33	Erlotinib	16	rs58643880	8.12	3_prime_UTR_variant	*NFAT5*	ENSG00000102908
34	Paclitaxel + Epirubicin	16	rs11639947	6.18	intron_variant	*NFAT5*	ENSG00000102908
35	Trametinib	16	rs11639947	8.11	intron_variant	*NFAT5*	ENSG00000102908
36	Trametinib	16	rs11641233	6.14	3_prime_UTR_variant	*NFAT5*	ENSG00000102908
37	Trametinib	16	rs12447326	6.08	intron_variant	*NFAT5*	ENSG00000102908
38	Trametinib	16	rs2361838	6.70	intron_variant	*NFAT5*	ENSG00000102908
39	Trametinib	16	rs58643880	7.08	3_prime_UTR_variant	*NFAT5*	ENSG00000102908
40	Arsenic trioxide	16	rs1437135	6.04	intron_variant	*NQO1*	ENSG00000181019
41	Erlotinib	16	rs1437135	8.31	intron_variant	*NQO1*	ENSG00000181019
42	Erlotinib	16	rs1800566	8.69	missense_variant	*NQO1*	ENSG00000181019
43	Paclitaxel + Epirubicin	16	rs1800566	6.15	missense_variant	*NQO1*	ENSG00000181019
44	Trametinib	16	rs1437135	6.64	intron_variant	*NQO1*	ENSG00000181019
45	Trametinib	16	rs1800566	8.07	missense_variant	*NQO1*	ENSG00000181019
46	Erlotinib	8	rs2444306	6.58	intron_variant	*OXR1*	ENSG00000164830
47	Temozolomide	3	rs4470517	6.15	intron_variant	*RYK*	ENSG00000163785
48	Temozolomide	3	rs4854617	6.47	intron_variant	*RYK*	ENSG00000163785
49	Fluoro-deoxyuridine	4	rs9994654	7.20	intron_variant	*SLC9B1*	ENSG00000164037
50	Fluoro-deoxyuridine	4	rs10516497	6.63	intron_variant	*SLC9B2*	ENSG00000164038
51	Docetaxel	5	rs2304035	6.76	missense_variant	*SLIT3*	ENSG00000184347

SNPs associated with the multivariate response for each drug at the genome-wide suggestive significance level or higher from the genome-wide association mapping using MAGWAS. The results are sorted by the host gene and drug. Chr: Chromosome, The most severe consequence obtained from Ensembl VEP (Ensembl release 97 –July 2019).

Variants in or close to the nuclear factor of activated T-cells 5 (*NFAT5)* and NAD(P)H quinone dehydrogenase 1 (*NQO1)* genes (located next to each other on chromosome 16) had the most consistent association with response across 44 anticancer treatments. Six unique SNPs in or close to the *NFAT5* gene were associated with response to erlotinib, trametinib, and the paclitaxel + epirubicin combination treatment, and two unique SNPs in the *NQO1* gene were associated with drug response to one or more of arsenic trioxide, erlotinib, trametinib, and the paclitaxel + epirubicin combination treatment (Table 2).

**Fig 1:**
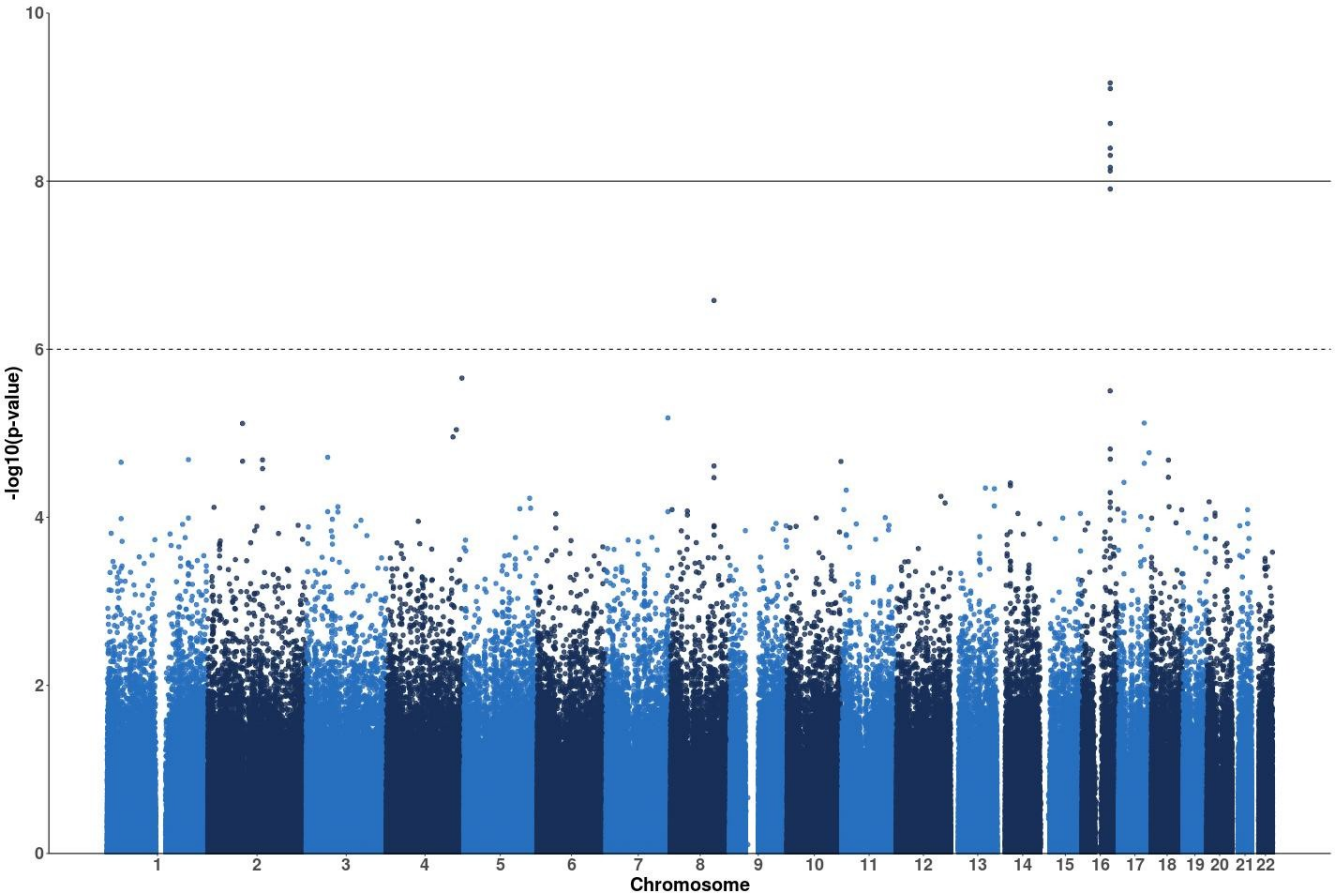
Manhattan plot of MAGWAS p-values for the drug erlotinib. Manhattan plot of MAGWAS -log_10_ (p-value) over 22 autosomes for the association of genotype and cell viability for the drug erlotinib. The dashed and solid lines indicate the thresholds for the genome-wide suggestive significance level of 10^−6^ and the genome-wide significance level of 10^−8^, respectively. The peak on chromosome 16 has seven SNPs surpassing the genome-wide significance level (the solid horizontal line), including the SNPs rs12447326 and rs1800566.

**Fig 2:**
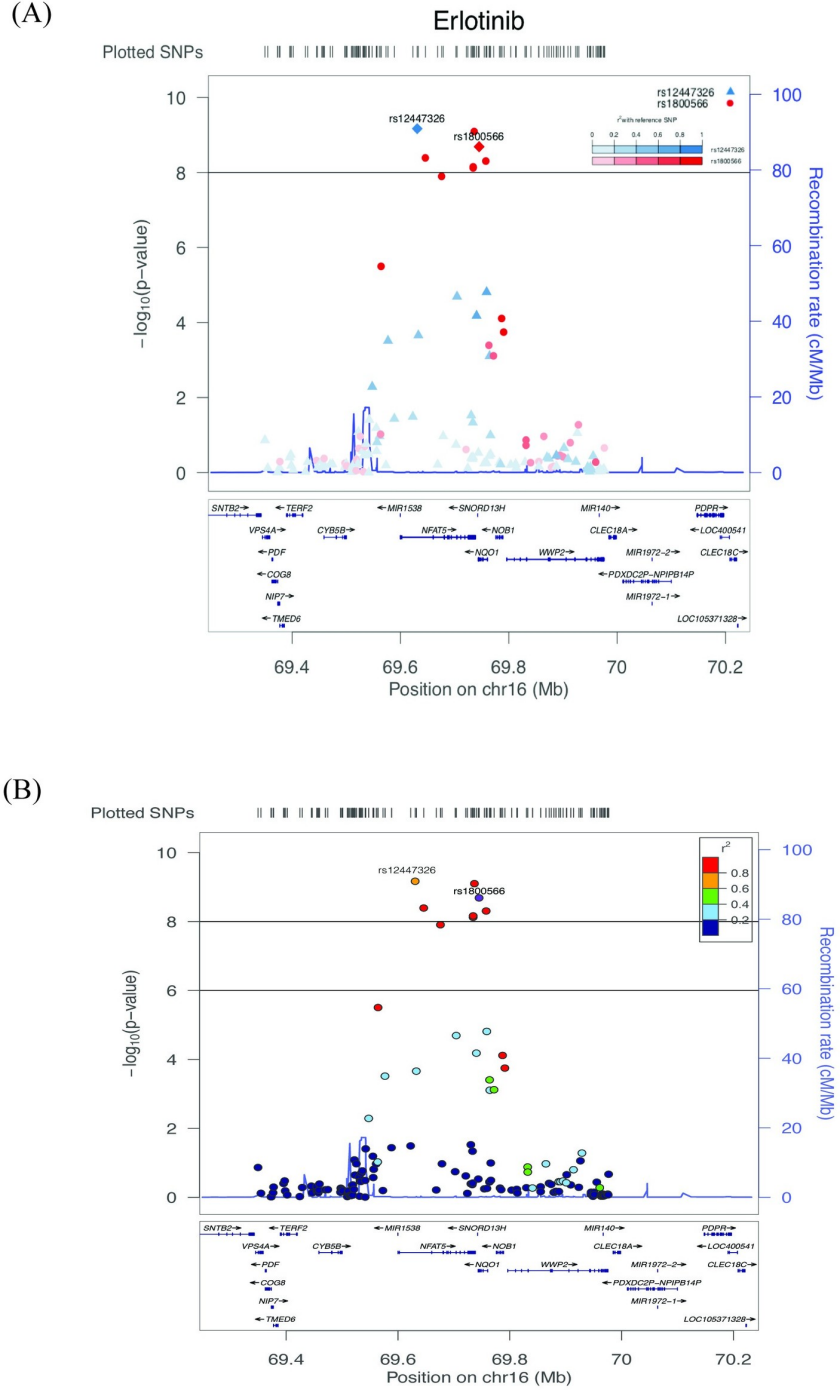
LocusZoom plot of the genes surrounding SNP rs1800566 on chromosome 16 for the drug erlotinib. LocusZoom plots showing the regional genes surrounding a 1 mega base pair region around SNP rs1800566 on chromosome 16 for associations with erlotinib. In the top panel, SNPs rs12447326 and rs1800566 are both used as lead/reference SNPs (shown as diamonds). For all other non-lead SNPs (shown as circles and triangles), their color and shape are matched to the lead SNP with which it is in the highest linkage disequilibrium (LD), as shown in the legend. The extent of LD with the lead SNP is shown by the color gradient. In the bottom panel, SNP rs1800566 is used as the lead SNP, and all other SNPs are colored according to their level of LD with rs1800566, as shown in the legend.

The most significant association was for SNP rs12447326 (p-value = 6.78e^-10^) in the *NFAT5* gene on chromosome 16 with response to erlotinib. Fig 1 shows a Manhattan plot for associations of SNPs with response to erlotinib. Manhattan plots for the other drug treatment GWAS are included in Fig A in S1 Text. The chromosome 16 peak centered on the *NFAT5/NQO1* locus has seven SNPs that surpass the genome-wide significance level (the solid black horizontal line), including sentinel SNPs for each gene. Fig 2A and 2B show zoomed-in regional gene views of this peak for associations with the drug erlotinib. Fig B in S1 Text shows regional gene plots for the same peak for associations with arsenic trioxide, trametinib, and the paclitaxel + epirubicin combination. rs12447326, which has been previously associated with a 40% reduced risk for rectal cancer [35], in the *NFAT5* gene was significantly associated with response to erlotinib and trametinib. The *NFAT5* gene encodes a transcription factor that regulates the expression of genes induced by osmotic stress [36] and has been implicated in other biological roles such as embryonic development, cell migration, and cell proliferation [37, 38]. *NFAT5* and *NFAT5* target genes have been reported to have very high expression in renal carcinoma cells and knockout *NFAT5* decreased proliferation and migration of these cells [37]. In addition, *NFAT5*-deficient lymphocytes have been observed to have decreased proliferation and viability when exposed to hypertonic stress [39].

**Fig 3:**
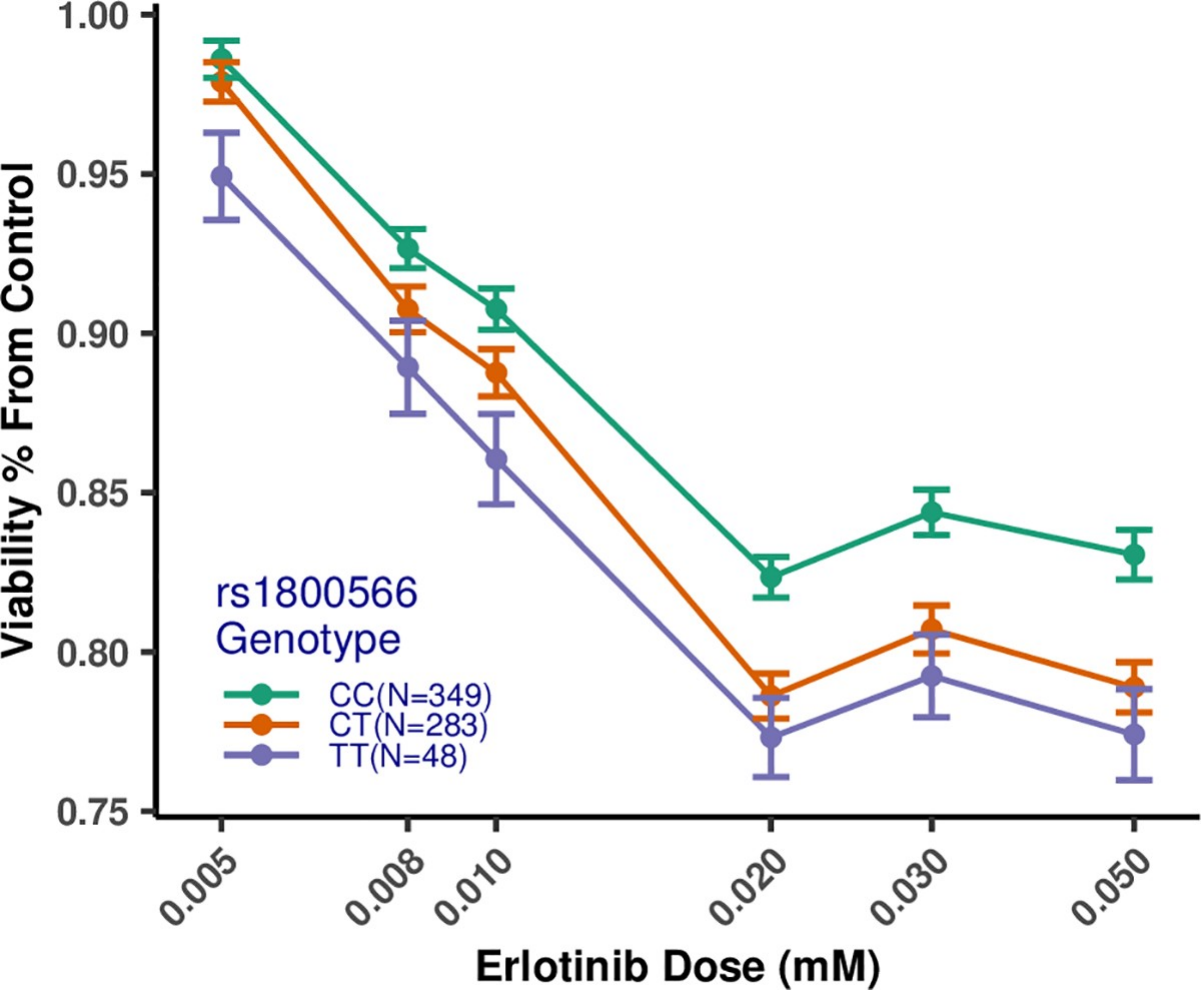
Dose-response profiles for erlotinib stratified by genotype at SNP rs1800566. C is the reference allele, and T is the variant allele at SNP rs1800566. Individuals with the TT (alternate) genotype have lower cell viability than those with the CC (reference) genotype. Heterozygous individuals (i.e., those with the CT genotype) have intermediate cell viability compared to CC and TT individuals. The numbers of individuals for each genotype are: CC—349, CT—283, TT—48. Concentrations are on the log10 scale on the X-axis. Bars represent the standard error of the mean.

We report that SNP rs1800566 is significantly associated with response to three drug treatments (i.e., erlotinib, trametinib, and paclitaxel + epirubicin). This coding SNP is characterized as a missense mutation, which is a C to T substitution at position 609 of the *NQO1* cDNA, and codes for a proline to serine change at position 187 of the amino acid structure of the enzyme [40]. This variant of the *NQO1* protein is unstable and rapidly degraded by the ubiquitin proteasomal system [41, 42]. Lymphoblastoid cells from individuals with two alternate alleles at rs1800566 (TT genotype) were more sensitive to erlotinib toxicity compared to heterozygous individuals (CT genotype) and homozygous reference individuals (CC genotype) (Fig 3). Similar dose-response profiles were seen for trametinib and paclitaxel + epirubicin combination treatment for rs1800566 (Figs D and E in S1 Text). Additionally, we conducted a multivariate GWAS controlling for SNP rs1800566 and did not find any SNPs significantly associated with response to any of the drugs in this genomic region (Fig C and Table B in S1 Text), motivating us to pursue functional experiments to further investigate this association.

### Gene-expression analyses results

To further interrogate the associations of *NFAT5* and *NQO1* with drug response, we tested for association between basal gene expression and cytotoxic drug response. RNA-Seq expression data is publicly available from the Geuvadis project for 272 of the LCLs in the current study [43]. We first performed extensive quality control on the RNA-Seq expression data to remove technical replicates, normalize for library depth, and correct for overdispersion [44, 45]. To characterize the correlation of baseline (non-drug-exposed) gene expression with cell viability, we tested for associations between basal gene expression of *NFAT5* and *NQO1* (using the quality-controlled RNA-Seq data) and cell viability across all treatment responses, using a two-stage multivariate linear regression model with the RNA-Seq lab and the sex of the individual, and the first three principal components as covariates. Table 3 shows significant results after a Bonferroni correction with a significance level of p < 0.05 applied per drug.

**Table 3:** Significant results from the multivariate linear regression of drug response on the baseline expression of *NQO1* and *NFAT5* genes.

Gene symbol	Drug	p-value	Bonferroni corrected p-value
*NQO1*	Arsenic trioxide	0.00066	0.00132
*NQO1*	Etoposide	0.00283	0.00566
*NQO1*	Sorafenib	0.00368	0.00737
*NQO1*	Temozolomide	0.00591	0.01183
*NQO1*	Cytosine beta-D-arabinoside	0.01173	0.02346
*NQO1*	Teniposide	0.01907	0.03814
*NFAT5*	Sorafenib	0.00795	0.01590

Significant correlations between the multivariate drug response and the baseline expression of *NQO1* and *NFAT5* genes after a Bonferroni correction with a significance level of p < 0.05 applied per drug.

Except for nintedanib, baseline gene expression of *NQO1* was positively correlated with response for all drugs at all concentrations. Thus, individuals with higher baseline *NQO1* expression had higher cell viability when exposed to all drugs except nintedanib, (i.e., higher resistance to the drugs) at all concentrations. To the best of our knowledge, we are the first to report this positive correlation of baseline *NQO1* expression with increased drug resistance for a wide range of anticancer agents. Thus, we hypothesize that *NQO1* may be involved in the drug-response pathways of multiple anticancer agents.

Additionally, we processed the RNA-Seq data to quantify transcript-level expression. We repeated the association analysis testing for the relationship between basal expression and dose-response for each transcript in *NFAT5* and *NQO1*. The results of these analyses align with our previous results and show that the expression of specific transcripts of *NFAT5* and *NQO1* are significantly correlated with the response of some of the drugs with significant GWAS SNPs in these genes (Table C in S1 Text).

### Cancer cell dependency analysis

To investigate whether GWAS-significant genes are essential in human cancer cell lines, we examined gene essentiality data obtained from genome-scale CRISPR-Cas9 genetic perturbation for 17,395 human genes across more than 900 cell lines, from the Cancer Dependency Map (DepMap) project [46, 47]. Our analysis showed significant changes in gene expression for 15 out of the 18 GWAS-significant genes with no data available for *MKX-AS1*, *LINC01081*, and *GMDS-DT*. Expression changes were split evenly with *NFAT5*, *NQO1*, *SLC9B2*, *OXR1*, *RYK*, *MRGBP*, and *AGAP3* upregulated and *SLC9B1*, *ITIH5*, *SLIT3*, *CACNB4*, *CREB5*, *CPED1*, *CSMD1*, and *ADRA1A* downregulated across the cell lines. In addition, we found that only two genes, *SLC9B1* and *MRGBP*, showed cell line dependence in 402 and 754 cell lines, respectively, while the copy number was one for all genes.

We evaluated the correlation between GWAS-significant gene expression and drug sensitivity patterns across 578 cell lines in the PRISM database [48] and 481 compounds across 823 cell lines in the CTD^2^ [49, 50]. We found a significant positive relationship between *NQO1* and etoposide, and *AGAP3* and vandetanib, as well as a significant negative relationship between *NQO1* and trametinib as measured using Pearson correlation coefficients. Results for *NFAT5* were inconsistent between the dataset with no significant relation shown in PRISM and CTD^2^ showed a significant negative relationship for trametinib exposure. Interestingly, no data were available in either of these databases for eight gene-drug combinations (hydroxyurea-*ADRA1A*, vemurafenib-*GMDS-DT*, vinblastine-*LINC01081*, oxaliplatin-*MKX-AS1*, arsenic trioxide-*NFAT5*, arsenic trioxide-*NQO1*, paclitaxel + epirubicin-*NFAT5*, paclitaxel + epirubicin-*NQO1*).

### *NQO1* protein quantitative trait locus (QTL) analysis results

**Fig 4:**
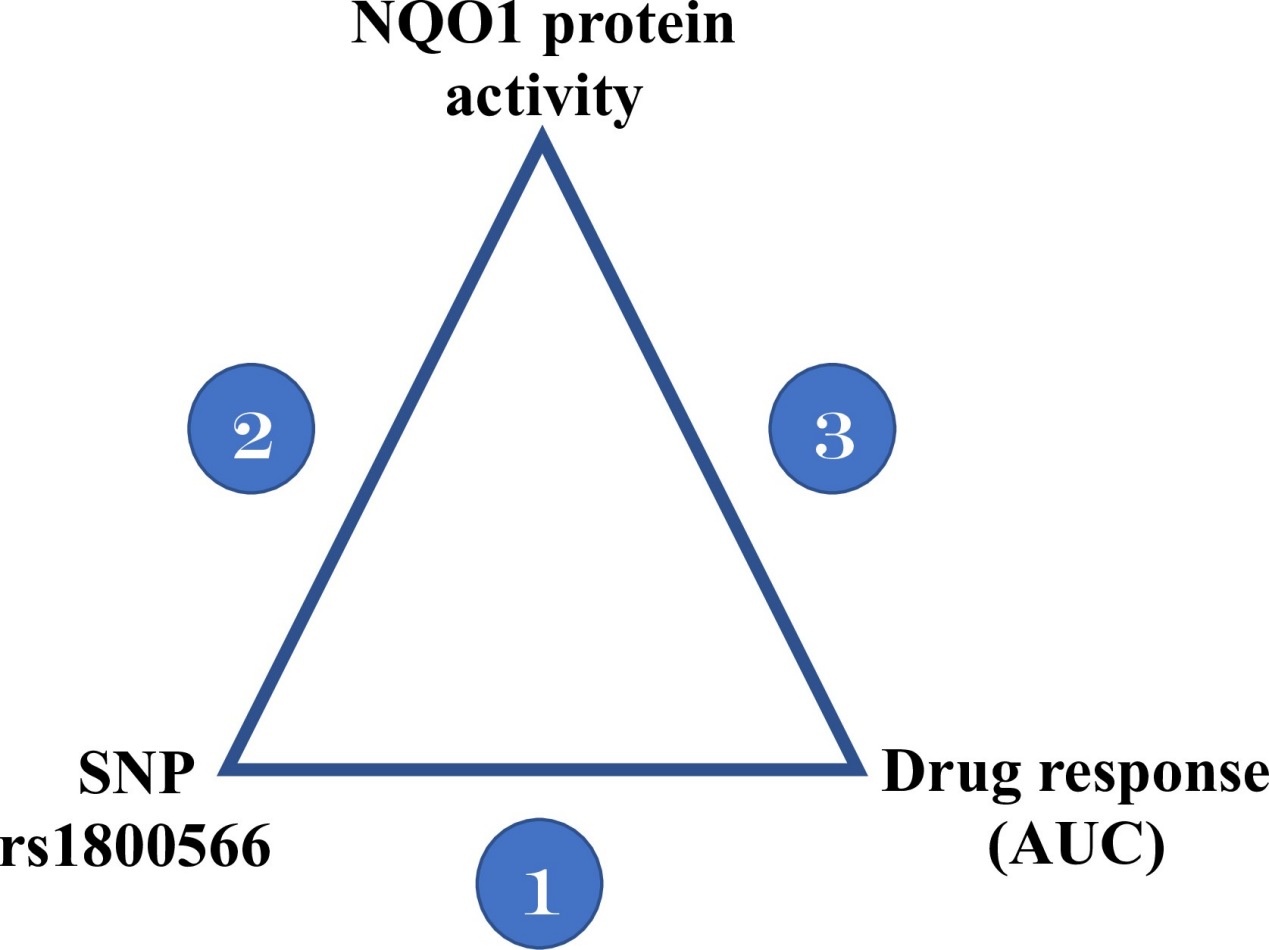
The three-stage protein QTL model used to identify the mechanism by which SNP rs1800566 influences drug response. Stage 1: the association between SNP rs1800566 and drug response measured as AUC; Stage 2: the association between SNP rs1800566 and NQO1 protein activity; and Stage 3: the association between NQO1 protein activity and drug response.

**Fig 5:**
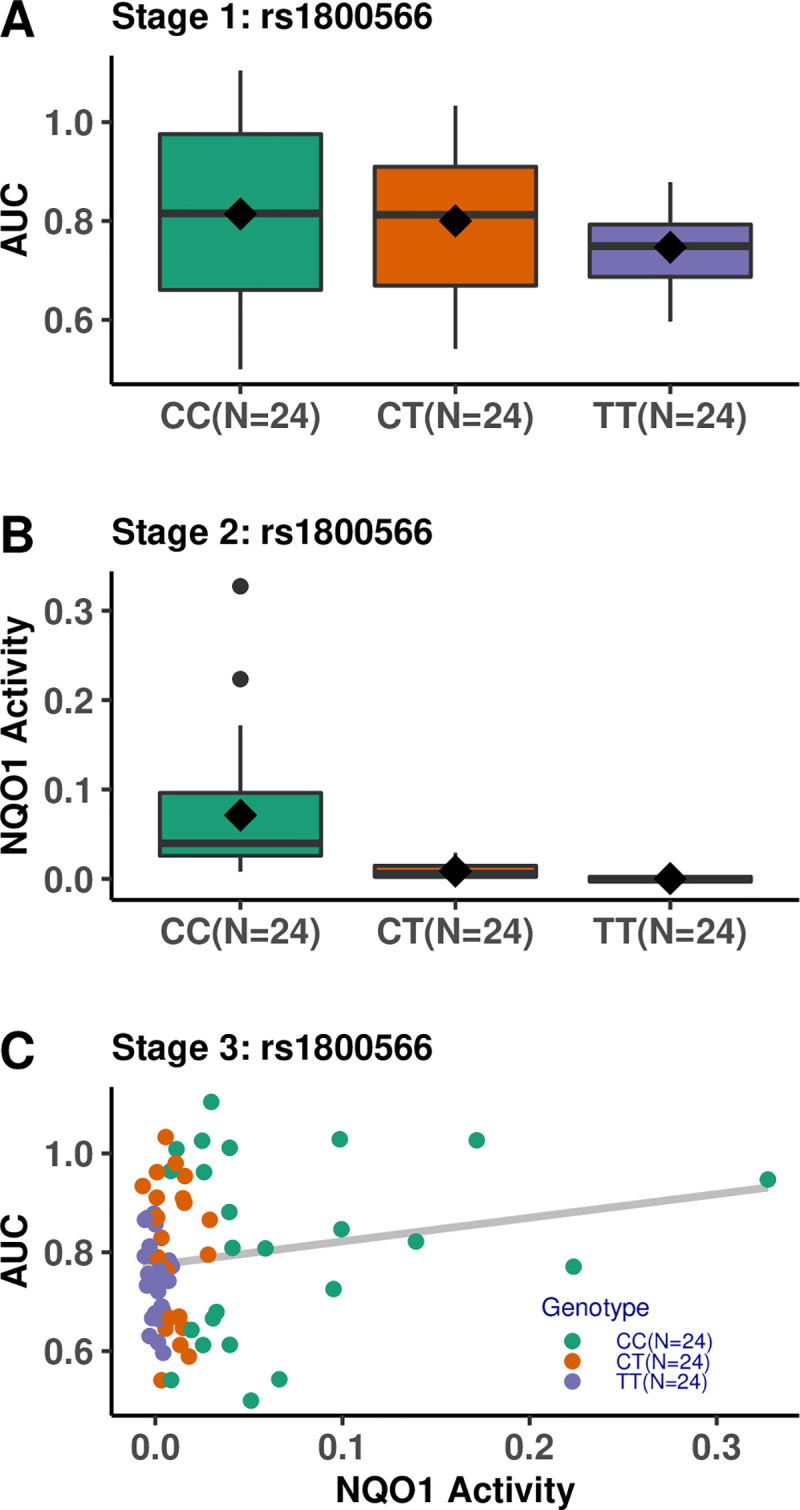
Associations for the pQTL model between genotype at rs1800566, NQO1 protein activity, and arsenic trioxide response data in 72 LCLs from the 1000 Genomes Project. (A) Stage 1: Association between SNP rs1800566 and AUC for arsenic trioxide. (B) Stage 2: Association between SNP rs1800566 and NQO1 protein activity. (C) Stage 3: Association between NQO1 protein activity and AUC for arsenic trioxide.

SNP rs1800566, the missense variant identified by GWAS mapping, alters the stability of the *NQO1* protein and is associated with drug-response variability to mitomycinC, β-lapachone, and epirubicin [51–58]. We hypothesize that this is also the mechanism by which rs1800566 influences the dose-response phenotype for the drugs in our assay; to test this, we used a three-stage protein QTL (pQTL) model. For rs1800566, we analyzed the correlations between genotype and *NQO1* protein activity (measured from the NQO1 Activity Assay Kit (ab184867) from Abcam (Cambridge, UK)) and genotype and drug-response data (area under the curve (AUC)) for the four drug treatments (i.e., arsenic trioxide, erlotinib, trametinib, and paclitaxel + epirubicin combination) as well as *NQO1* protein activity and drug-response data for 24 LCLs of each genotype (CC (reference) vs. CT vs. TT) (Fig 4). Table D in S1 Text summarizes the results, and Fig 5 displays the arsenic trioxide results as an example.

The first stage of the pQTL model shows that the AUCs for arsenic trioxide and erlotinib are significantly associated with the genotype at rs1800566 (Fig 5A). Stage 2 reveals that *NQO1* activity was highly associated with the genotype (p = 5.27e-10), and individuals with the TT genotype had markedly decreased *NQO1* activity compared to those with the CC (reference) genotype (Fig 5B). Finally, in Stage 3, *NQO1* enzyme activity was associated with the arsenic trioxide AUC (Fig 5C). Associations for the other three drug treatments were not significant. Overall, the pQTL analysis provides some evidence that SNP rs1800566 influences drug response by altering *NQO1* protein activity for the four aforementioned drug treatments. The pQTL model was statistically significant for arsenic trioxide at all three stages (Table D in S1 Text) but may have been underpowered to detect statistical significance for other drug treatments.

We repeated the third stage of the model stratified by the genotype at rs1800566 (Table E in S1 Text). Interestingly, this stratified analysis showed that while *NQO1* activity was positively correlated with the AUC for individuals with the CC and CT genotypes, it was negatively correlated for individuals with the TT genotype at SNP rs1800566 for all drug treatments. This suggests a different relationship between drug response and *NQO1* protein activity in individuals with different genotypes at rs1800566.

### Additional Fine Mapping in *NQO1*

To further interrogate the GWAS signal, additional sub-threshold SNPs in linkage disequilibrium with rs1800566 were tested for association with AUC as described above in the third stage of analysis. An additional SNP, rs689457, was statistically significant for several of the GWAS-flagged drug treatments, with the strongest signal for trametinib. rs689457 is approximately -870bp from the transcriptional start site of the gene, and follow-up experiments show that variants affect *NQO1* expression (Fig G in S1 Text). To examine the effect of this SNP in a more controlled, genetically matched background, the NQO1 promoter was placed upstream of GFP in a reporter plasmid and transfected into the same cell line in parallel with a wild type-based plasmid, with results shown in Fig G in S1 Text.

### Functional assay results

#### Reactive oxygen species assay results

The recurrence of *NQO1* in our analyses suggests oxidative stress is a pervasive off-target phenomenon. Accordingly, we examined which of the studied drugs could induce oxidative stress and estimated the relative levels among the various drug classes. We chose and examined together two immortalized cell lines (LCL NA19119 and kidney cell line HEK-293) and two solid tumor cell lines (melanoma cell line WM266-4 and breast carcinoma cell line MDA-MB-436) to develop a generalized picture of oxidative stress potential across multiple cell types. Each cell line was treated with the maximum concentration (of each compound) used in the viability screening experiments for 12 h to limit reactive oxygen species (ROS) generation from cell death. The fold change of induction of ROS over the controls was averaged across the four tested cell types (Table 4). Under these conditions, most compounds can induce ROS (Fig I in S1 Text). Surprisingly, the strongest ROS signals are from the tyrosine kinase inhibitors (TKI) class of drugs. While anthracyclines are known inducers of oxidative stress, their signal may be more dependent on cell cycling and would likely increase given longer treatment times before ROS assessment.

**Table 4:** Drug-induced oxidative stress results from ROS assays.

Drug class	Compound	ROS induction
Anti-metabolite	Hydroxyurea	-
DNA alkylating agent	MitomycinC	-
	Temozolomide	+
Combination treatment	Paclitaxel + Epirubicin	+
Epipodophyllotoxins	Teniposide	+
	Etoposide	++
Anthracyclines/anthracendiones	Daunorubicin	+
(Topoisomerase II inhibitors)	Doxorubicin	+
	Mitoxantrone	+
	Idarubicin	+
	Epirubicin	++
	Topotecan	++
Microtubule binding agents	Docetaxel	-
	Vinorelbine	++
	Vincristine sulfate	++
	Paclitaxel	++
	Vinblastine	+++
Nucleosides	Cytosine beta-D-arabinoside	+
	Gemcitabine	++
	Fludarabine	++
	Cladaribine	++
	Azacytidine	++
Fluoropyrimidines	5-Fluorouracil	+++
	Fluoro-deoxyuridine	+++
Other	Arsenic trioxide	++
Platinum agents	Oxaliplatin	++
	Carboplatin	+++
Tyrosine Kinase inhibitors	Sunitinib	-
	Masatinib	-
	Dasatinib	+
	Ibrutinib	+
	Axitinib	+
	Dovitinib	++
	Cabozantinib	+++
	Vandetanib	+++
	Erlotinib	+++
	Trametinib	++++
	Nilotinib	++++
	Sorafenib	++++
	Crizotinib	++++
	Tivantinib	++++
	Apatinib	+++++
	Nintedanib	+++++

ROS was measured for each drug in duplicate following 12 h treatment with study compounds, and the fold induction over control was determined. The results for the four cell-line types (NA19119-lymphoblastoid, WM2664-melanoma, MDA-MB-436-breast carcinoma, and HEK-293-kidney) were then averaged. Data are presented as: -, 0–1; +, ≤1.25; ++, ≤1.5; +++, ≤1.75; ++++, ≤2.0, and +++++, ≥2.0.

#### mRNA expression assays results

**Fig 6:**
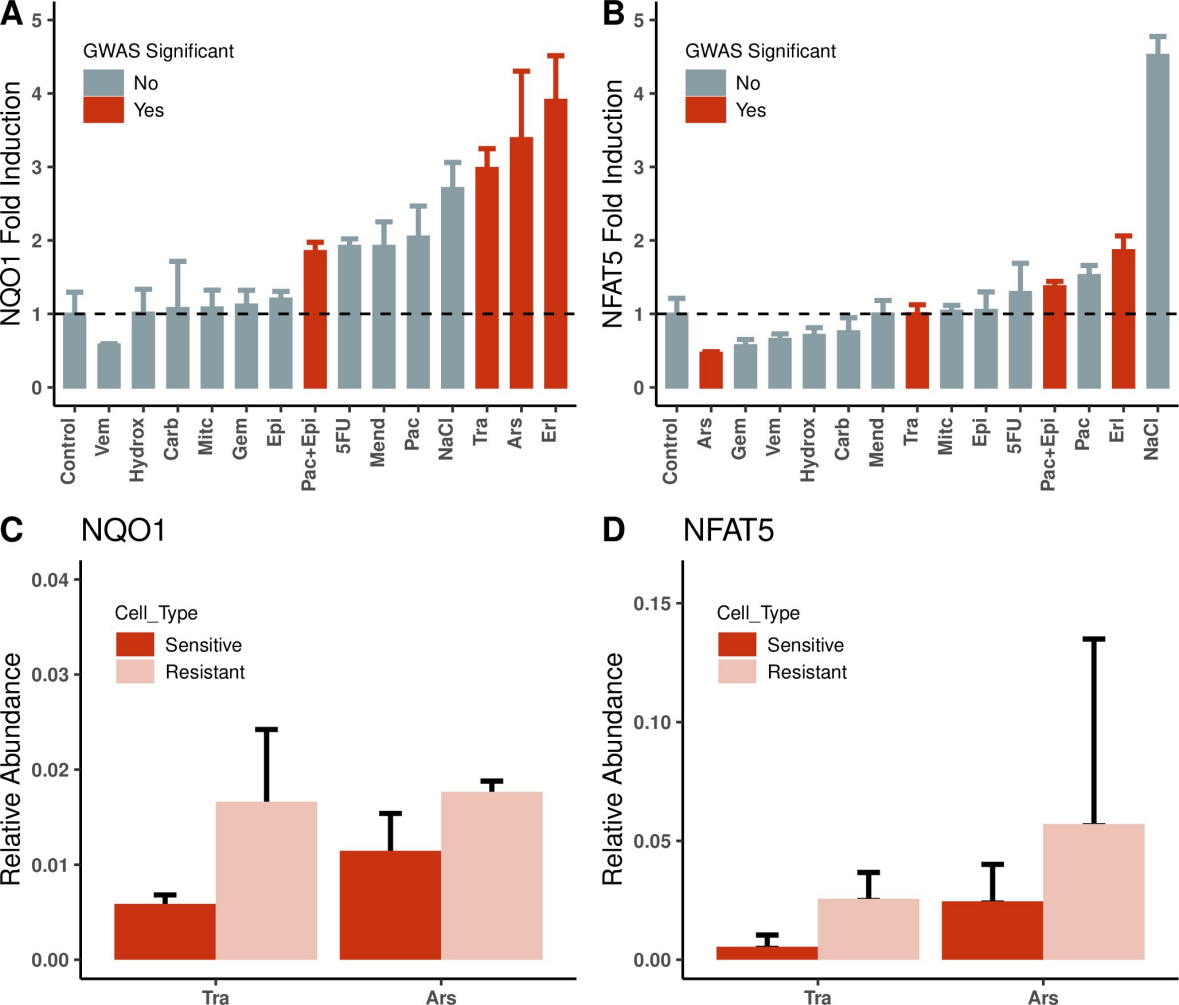
Drug-induced gene expression of *NQO1* and *NFAT5*. (A) & (B): Transcriptional activity of *NQO1* and *NFAT5* measured following 24 h treatment of an LCL (NA19119) with GWAS-flagged compounds (red bars) as well as compounds representative of the various drug classes (grey bars) at the maximum concentration used in the cell viability screening in this study. Menadione (5uM) and NaCl (90mM) were included as positive controls for *NQO1* and *NFAT5*, respectively. (C) & (D): Comparison of the average *NQO1* and *NFAT5* gene induction in sensitive versus resistant cell lines: The above experiment was repeated with the compound, arsenic trioxide (**Ars**), or the TKI, trametinib (**Tra**), using groups of six cell lines each, identified as sensitive (red bars) or resistant (pink bars). Drug treatments: - Ars:Arsen, Carb:Carboplatin, Epi:Epirubicin, Erl:Erlotinib, 5FU:5-Fluorouracil, Gem:Gemcitabine, Hydrox:Hydroxyurea, Mit:MitomycinC, Pac:Paclitaxel, Tra:Trametinib, Vem:Vemurafenib, Pac+Epi:Paclitaxel+Epirubicin combination treatment, Mend:Menadione, NaCl:Sodium Chloride.

Since baseline gene expression of *NFAT5* and *NQO1* was found to be positively correlated with cell viability for the majority of drugs, we examined whether induced gene expression could distinguish compounds with the strongest GWAS results from the remaining test drugs. GWAS-flagged compounds were compared against a subset of compounds representative of the various drug classes studied. We treated cell line NA19119 with the half-maximal treatment dose and measured gene expression by reverse transcriptase real-time quantitative polymerase chain reaction (PCR) after 24 h. Expression of *NQO1* tended to be associated with treatments with the highest GWAS significance (Fig 6A and Table 2). This trend was less evident with *NFAT5* (Fig 6B).

**Fig 7:**
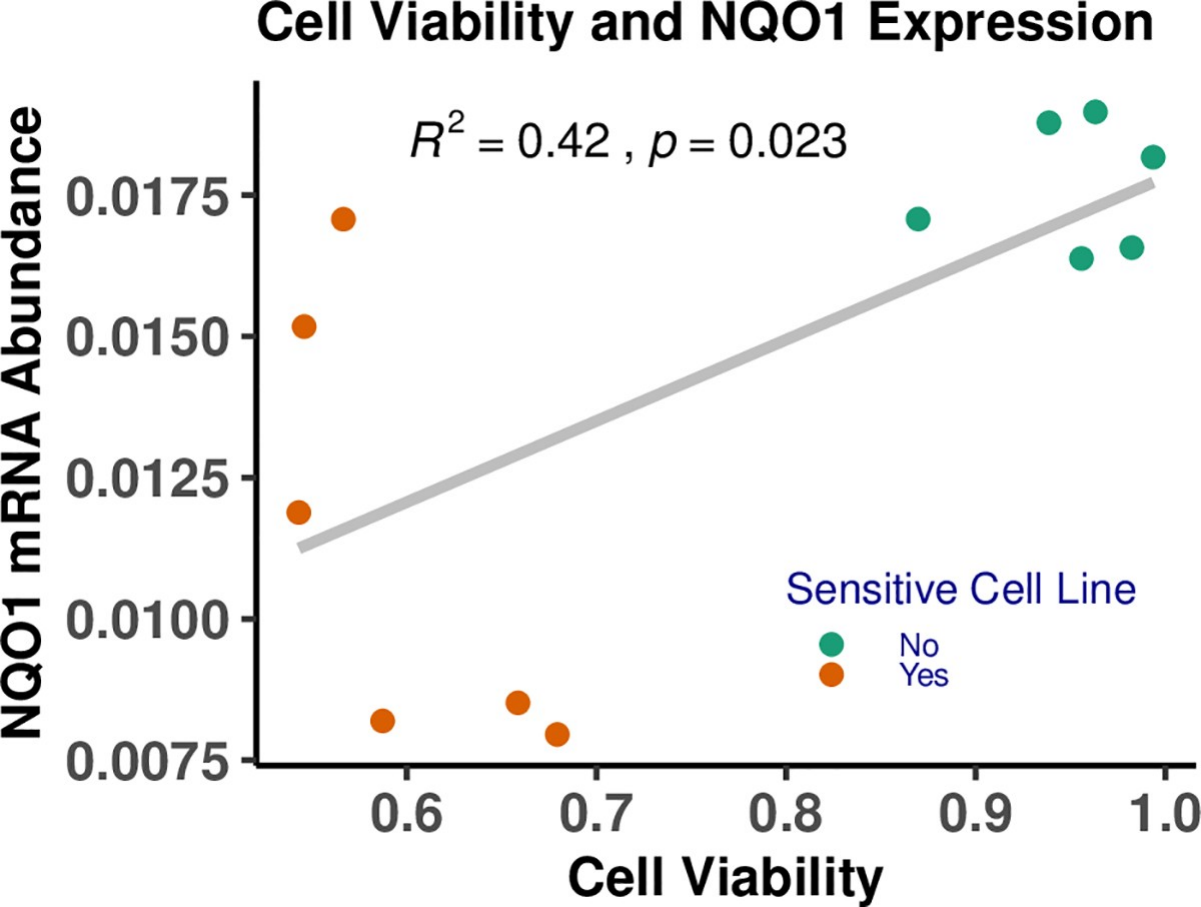
Correlation of *NQO1* mRNA expression and cell viability. Increased cell viability was observed with higher levels of *NQO1* mRNA expression as measured in 12 LCLs exposed to 0.5μM arsenic trioxide. The 12 selected LCLs comprise six sensitive and six resistant cell lines, classified based on their mean viability (AUC values) across the four drug treatments: arsenic trioxide, erlotinib, trametinib, and the paclitaxel + epirubicin combination. Each cell sample was measured in triplicate. The replicates were averaged for each cell line.

We subsequently examined *NQO1* and *NFAT5* gene expression for groups of sensitive or resistant cell lines (as determined from the observed cell viability in the drug-response assays) to determine if gene expression predicts cell response. We ranked cell lines by sensitivity or resistance based on the AUC of the response to the four GWAS-flagged treatments. We then compared basal and drug-induced levels of *NQO1* and *NFAT5* between sensitive and resistant groups following treatment with either arsenic trioxide or trametinib, a TKI. We chose arsenic trioxide and trametinib for these functional assays because of the observation of significant associations for these drugs in more than one of our previous analyses. Using a small sample size of six cell lines in each group, there was no statistically significant difference in baseline expression of *NQO1* and *NFAT5* between sensitive and resistant cell lines, but there were significant differences for induced expression, with resistant cell lines expressing significantly more *NQO1* than sensitive cell lines (Fig 6C). Induced *NFAT5* expression tended to parallel the results for *NQO1*, but induction relative to control was modest and not significant for arsenic trioxide (Fig 6D). This differential expression is significantly associated with cell viability, as shown for arsenic trioxide in Fig 7.

To summarize, drug treatments associated with GWAS hits were also associated with increased *NQO1* expression. Further, *NQO1* expression and cell viability were positively correlated in cell lines exposed to the drugs flagged for *NQO1* in GWAS. Fig 7 shows the related results for arsenic trioxide. We also examined *NQO1* expression in the most sensitive and most resistant cell lines in our assays for each drug flagged for *NQO1* by GWAS. Resistant cell lines tended to exhibit increased expression of *NQO1* compared to sensitive cell lines (Fig 6C and 6D). Collectively, these assays confirm our hypothesis that *NQO1* plays an important role in the drug-response pathways of multiple anticancer agents.

### Overall results

Collectively, our high-throughput GWAS screening identified a number of potential drug-gene associations, with 18 total GWAS hits across the drug treatments evaluated. Using extensive gene expression data from CRISPR screens across a number of cancer cell lines, we found gene expression changes in these GWAS hits are consistently associated with drug response from PRISM and CTD. This supports further biological plausibility of the associations and motivates further functional interrogation in future studies.

Within the current study, the results from the genome-wide association analysis, the gene and transcript expression analyses demonstrated that *NQO1* plays an important and consistent role in response to treatment with arsenic trioxide, erlotinib, trametinib, and the paclitaxel + epirubicin combination. We focused our functional follow-up on this top signal. To the best of our knowledge, *NQO1* has not previously been associated with response to arsenic trioxide or the paclitaxel + epirubicin combination. Through this study, we identified a novel association with multiple individual drug responses and with a synergistic drug combination. Further, the results from the pQTL analysis indicate a possible mechanism by which GWAS-significant SNP rs1800566 influences the response to these treatments. We identified an additional novel SNP rs689457 in *NQO1* also associated with multiple drug responses. From these results, we conclude that *NQO1*, an oxidative stress response gene, is involved in a common drug-response pathway(s) and plays a role in the inter-individual variation in response to these drugs, with higher *NQO1* expression and protein activity associated with increased resistance to the cytotoxic activity of these drugs.

## Discussion

In the current study, we present the results of the largest LCL screen of anticancer drugs to date. While most studies have assayed two to four drugs [59–62], in the current study, we assayed 179,520 drug-dose-cell line combinations (29,920 drug-cell line combinations) with a wide range of anticancer drugs spanning multiple drug classes. In total, we identified 51 suggestively significant SNP-drug associations at the genome-wide level using association mapping (Table 2). From these hypothesis-generating results, we followed up on the consistent signals in *NQO1* to better understand the functional role of this gene in drug response. Given the strong association of *NQO1-*related variants with multiple drug treatments (arsenic trioxide, erlotinib, trametinib, and a combination treatment of paclitaxel + epirubicin), we conducted follow-up experiments to better understand the functional role of this gene in anti-cancer drug response. Both increased *NQO1* expression and *NQO1* protein activity were associated with increased resistance to these drugs and suggest variation in *NQO1* may contribute to the inter-individual variation in response to the treatments via a common drug-response pathway(s). This is suggested through the role *NQO1* plays in oxidative stress response, and the stabilization of *TNF-α* and *P53*, which are important in tyrosine kinase inhibition and topoisomerase II inhibition, respectively [63]. These novel associations for *NQO1* suggest the potential use of *NQO1* expression levels as a biomarker to predict the variability in response to the individual anticancer drugs and combination treatment.

rs1800566 has previously been associated with mitomycinC, β-lapachone, and epirubicin drug response [56–58]. Further, *in vitro* arsenic trioxide treatment results in increased cell death and *NQO1* protein levels [4]. A lack of *NQO1* protein due to SNP rs1800566 is associated with many cancers, including adenocarcinoma of the gastrointestinal tract, gastric cardiac carcinoma, and esophageal, lung, bladder, and colorectal cancers [51–55]. *NQO1*, an antioxidant enzyme important in environmental carcinogen detoxification [50, 56], encodes a cytoplasmic 2-electron reductase and reduces quinones to hydroquinones. Several anticancer agents such as mitomycinC and β-lapachone are bioactivated by *NQO1*. Since *NQO1* is expressed at higher levels in many human solid tumors compared to normal tissue, this allows for the selective activation of these prodrugs in tumor cells [56, 57]. Higher levels of NQO1 have also been shown to sensitize cells to undergo apoptosis, which is the primary mechanism by which topoisomerase II inhibitors like etoposide act and provides mechanistic evidence for the positive correlation between etoposide exposure and *NQO1* gene expression [63, 64].

The overall results of our pQTL analysis provide evidence that SNP rs1800566 influences drug response by altering protein activity for the four aforementioned drug treatments. Our results supplement those of prior studies that have shown the phenotypic effect of the missense variant rs1800566 [46, 47, 56–58, 65, 66] by demonstrating that while the wildtype *NQO1* protein is associated with increased resistance, the variant protein resulting from the TT genotype is associated with increased sensitivity for the four drug treatments. While only the associations for arsenic trioxide and erlotinib are statistically significant in this model, power limitations due to reduced sample size (n = 72) in this analysis need to be considered in interpretation.

We have demonstrated that increased *NQO1* activity induces a drug-resistant phenotype, presumably with the ability to better manage the stress response of the cells. In addition to the missense variant rs1800566, we also report a novel SNP association in *NQO1* because our results indicate that rs689457 is putatively associated with the regulation of *NQO1* and thus affects drug response (Fig G in S1 Text).

As in any *in vitro* model, our results require further validation and replication in human studies [13, 20]. Further, while our integrative analysis methods identified a strong correlation between higher *NQO1* expression levels and resistance to the specified drugs, additional experiments are needed to identify the specific mechanism of action by which *NQO1* influences these responses. The genome-wide mapping identified six unique SNPs in the *NFAT5* gene, which is located in proximity to *NQO1* on chromosome 16, that are associated with dose-response. However, we were unable to conduct knockdown experiments to examine the effects of *NFAT5* on dose-response for two reasons: a) it is a transcription factor that regulates the expression of a large number of genes, and b) previous investigations of *NFAT5* have reported cell cycle arrest in *NFAT5*-null T lymphocytes [39] and severe impairment of cell proliferation in *NFAT5*-null mice [54] under hyperosmotic culture conditions. There was no significant difference in induced *NFAT5* expression in sensitive vs. resistant LCLs exposed to GWAS-flagged compounds (Fig 6D) and no significant correlation between *NFAT5* gene expression and GWAS-flagged drug treatments in cancer cell lines using the PRISM and CTD^2^ databases from DepMap. Following treatment with the GWAS-flagged drug treatments, qPCR assays measuring mRNA expression levels of *NFAT5* target genes S100 calcium-binding protein A4 (*S100A4*) and sodium/myo-inositol cotransporter (*SMIT*) showed no significant differences in expression of these genes (Fig H in S1 Text), with arsenic trioxide being the only exception. Thus, we did not conduct further functional assays to validate the role of *NFAT5* in the drug-response assays within the scope of this study.

Further studies are needed to translate to clinical practice our results indicating the possibility of using *NQO1* expression levels as a biomarker to predict drug resistance. Previous studies have reported that high levels of *NQO1* expression are associated with poor prognosis of breast cancer [67], gastric adenocarcinoma [68], and malignancy in pancreatic cancer [69]. This is consistent with our finding that higher *NQO1* expression is correlated with increased drug resistance. Altering *NQO1* expression potently triggers innate sensing within the tumor microenvironment, causing *NQO1*-activated β-lapachone to overcome immunotherapy resistance [70]. Given our results, we hypothesize that with further research, *NQO1* may be an important biomarker to guide dual therapies in general, with various drug classes as well as immunotherapies, to increase sensitivity or overcome drug resistance.

While we focus on the *NQO1* results in the current functional follow-up, our high-throughput screening revealed additional potential associations that should be followed up in future studies. At the suggestive significance level, 18 genes were identified across treatments, including several associated with known cancer pathways. For example, the GWAS results indicated that SNP rs17142881 was significantly associated with response to gemcitabine, which is used to treat breast cancer. rs17142881 is in the inter-alpha-trypsin inhibitor heavy chain family member 5 (*ITIH5*) gene, which acts as a tumor suppressor in breast cancer cell lines through epigenetic reprogramming and as a metastasis suppressor in breast and pancreatic cancers [71, 72]. Further, response to temozolomide was associated with rs4854617 and rs4470517 in the receptor-like tyrosine kinase (*RYK*) gene on chromosome 3. *RYK* is one of the receptors for Wnt family member 5A (*WNT5A*) and has been reported to be involved in invasive activity in glioma-derived cells [73–75] and to facilitate the pro-apoptotic and anti-proliferative effects of *WNT5A* in prostate cancer cells [76]. Further, response to carboplatin was associated with rs6010746 in the MRG domain binding protein (*MRGBP*), which has been identified as a potential biomarker for malignancy in pancreatic ductal adenocarcinoma [77] and reported to contribute to colorectal carcinogenesis by promoting cell proliferation in cancer cells [78]. The DepMap analysis showed a significant correlation between *AGAP3* expression and cancer cell line sensitivity to vandetanib, and that *AGAP3* is overexpressed in more than 98% of the 1,376 cancer cell lines in the database merits further investigation.

In this study, we present high-throughput *in vitro* screening data and genome-wide analyses results in LCLs for 44 anticancer drug treatments, including the combination paclitaxel + epirubicin treatment. We identify multiple genetic variants associated with the response to several drug treatments, including the combination paclitaxel + epirubicin treatment, indicating that potential biomarkers for synergistic drug response can be identified in LCLs. Our integrative analyses show that *NQO1*, an oxidative stress response gene, is associated with response to treatment with arsenic trioxide, erlotinib, trametinib, and the paclitaxel + epirubicin combination. The large-scale, systematic results of this study can serve as a valuable resource for future dose-response studies for a broad range of drugs widely used for the treatment of various types of cancers.

## Materials and methods

### Cell lines and genotypic data

We used 680 immortalized LCLs derived from the 1000 Genomes Project [30] that represent nine geographically and ethnically diverse populations: Utah residents with European ancestry (CEU); Han Chinese in Beijing, China (CHB); Japanese in Tokyo, Japan (JPT); Luhya in Webuye, Kenya (LWK); Residents of Los Angeles, California with Mexican ancestry (MXL); Tuscans in Italy (TSI); Yoruban in Ibadan, Nigeria (YRI); British from England and Scotland (GBR); and Colombian in Medellin, Colombia (CLM). Genotype data from the Illumina HumanOnmi2.5 platform was downloaded and processed as described in Abdo et al. [32]. Briefly, as part of quality control, we removed the SNPs with a call rate < 95%, minor allele frequency (MAF) < 0.01, or HWE p-value < 1 x 10^−6^, yielding 1,510,701 SNPs used for GWAS. We chose the subset of 680 cell lines from the available 1,086 1000 Genomes cell lines, after removing first-degree relatives using genotypes and sample annotation.

### Dose-response assays

Each drug was assayed at six concentrations. Each cell line was seeded on two 384-well plates with 4,000 cells per well and four replicates per plate. Each plate included a control for background fluorescence signal, 10% dimethyl sulfoxide (DMSO), and another control for drug vehicle, either water or DMSO at 0.1%, with the exception of temozolomide, which had DMSO at 0.08%, 0.20%, 0.41%, 0.62%, 0.82%, and 1.23%. Each cell line was incubated for 72 h with each of the treatments for all tested concentrations, dyed with alamarBlue (BioSource International), and incubated for another 24 h. The alamarBlue assay is a fluorometric and colorimetric cell viability assay incorporating an oxidation-reduction indicator that responds to cellular metabolic reduction, with the intensity of fluorescence produced proportional to the number of living cells. Subsequently, a Tecan Freedom EVO 150 robotics system with a Connect stacker and F200 plate reader, which measures fluorescence intensity in raw fluorescence units (RFUs), was used for viability measurements. We applied quality control procedures and calculated cell viability as previously described [1, 22, 29].

### Multivariate genome-wide association analyses (MAGWAS)

To identify common genome-wide SNPs associated with variability in drug response, we performed MANCOVA using MAGWAS software [32]. MAGWAS incorporates multivariate phenotypes, in this case, an entire dose-response profile instead of a univariate dose-response summary (e.g., IC_50_). These multivariate methods are more powerful than methods that employ a univariate response [13, 29, 32]. The model used for the association analysis for each drug at SNP *s* is:

$$Y_{ij}=X_{ij}\beta +\mu_{i}+e_{ij},e_{ij}∼N(0,\Sigma )$$

where Y_ij_ is the vector of normalized responses across the six concentrations of the drug for the *jth* individual with genotype *i* at SNP *s*; **X**_ij_ is the covariate matrix, which includes temperature, experimental batch, and the Eigenvalues from the first three principal components calculated using EigenStrat [79]; and μ_i_ is the vector of parameters modeling the effects of genotype *i* at SNP *s*. Further details of this analysis are included in Figs A and F and Table B in S1 Text [80–82].

### Gene-expression analyses

We obtained the RNA-Seq read-count data from the Geuvadis project [43] for 272 individuals in our assays. We performed extensive quality control to remove technical replicates, normalize for library depth, and correct for overdispersion [44, 45]. We examined the correlation of baseline gene expression for *NQO1* and *NFAT5* with dose-response using a two-stage multivariate linear regression model specified as follows:

- First stage: G_i_ = β_0_ + **L**_i_*β + ϵ_i_, ϵ_i_ ~ N(0, σ^2^)
- Second stage: ***Y***_*ij*_ = *β*_0_ + *β*_1_*PC1_*i*_ + *β*_2_*PC2_*i*_ + *β*_3_*PC3_*i*_ + *β*_4_*Sex_*i*_ + *β*_5_*ϵ_i_ + ***e***_***ij***_, ***e***_***ij***_ ∼ *N*(**0,Σ**)

where, in the first stage: G_i_ is the quality-controlled RNA-Seq read count of gene *g* for individual *i*, L_i_ is a vector of indicator variables for the lab in which the RNA-Seq was conducted for individual *i*, β is a vector of the regression parameters, and ϵ_i_ are the residuals = observed read count for gene *g*—predicted gene read count for genes for individual *i*; in the second stage: Y_ij_ is the vector of normalized responses for the six concentrations of the drug for individual *i*; PC_1_, PC_2_, and PC_3_ are the Eigenvalues from the first three principal components calculated using EigenStrat [79]; and sex is an indicator variable denoting the sex of individual *i*. Further details of this analysis are included in Figs G and H and the Gene Expression Two-stage Regression section in S1 Text.

Additionally, we conducted transcript expression analyses as described for the gene-expression analyses, using the expression for the multiple transcript isoforms instead of the single gene.

### Cancer cell dependency analysis

To determine which GWAS-significant genes exhibit essentiality, changes in copy number, or changes in gene expression across multiple human cancer cell lines, we utilized the DepMap portal (https://depmap.org/portal/). This portal contained the results of CRISPR-Cas9 knockout screens for 18,333 genes in 739 cancer cell lines along with RNA sequencing results from over 1,376 cell lines. In addition, we explored the correlation between gene expression and sensitivity patterns of 4,518 drugs tested across 578 cell lines (PRISM) and 481 compounds tested across 823 cell lines (CTD^2^).

### *NQO1* protein QTL analysis

To identify the mechanism by which our GWAS-significant SNP rs1800566 influences drug response for the four drug treatments, we conducted a three-stage protein QTL (pQTL) analysis as shown in Fig 4. For this analysis, 24 LCLs for each genotype at rs1800566 (reference = CC, heterozygous = CT, alternate = TT) were randomly selected from the 680 LCLs, for a total of 72 LCLs (sample size based on power calculations). In this analysis, we used the genotype data (from the 1000 Genomes Project), dose-response data (measured as described in the *Dose-response assays* section), and the *NQO1* protein activity data (measured as described in the *NQO1 enzyme activity assays* section) in the three-stage model specified as follows:

- Stage 1: AUC_*i*_ = *β*_0_ + *β*_1_*PC1_*i*_ + *β*_2_*PC2_*i*_ + *β*_3_*PC3_*i*_ + *β*_4_*Sex_*i*_ + *β*_5_* rs1800566_i_ + e_ij_, e_ij_ ∼ *N*(0, σ^2^)
- Stage 2: NQO1_protein_activity_i_ = *β*_0_ + *β*_1_*Sex_*i*_ + *β*_2_*rs1800566_i_ + e_ij_, e_ij_ ∼ *N*(0, σ^2^)
- Stage 3: AUC_*i*_ = *β*_0_ + *β*_1_*PC1_*i*_ + *β*_2_*PC2_*i*_ + *β*_3_*PC3_*i*_ + *β*_4_*Sex_*i*_ + *β*_5_*NQO1_protein_activity_i_ + e_ij_, e_ij_ ∼ *N*(0, σ^2^)

where AUC is the area under the curve; PC1, PC2, and PC3 are the Eigenvalues from the first three principal components calculated using EigenStrat [79]; sex is an indicator variable denoting the sex of individual *i*; rs1800566 is the number of minor alleles at that SNP; and NQO1_protein_activity is the baseline *NQO1* protein activity measured for each cell line using the NQO1 Activity Assay Kit (ab184867) from Abcam (Cambridge, UK). Details of this three-stage analysis are included in Tables D and E and the Protein QTL Analysis section in S1 Text.

### Functional assays

#### ROS assays

We conducted ROS assays in four cell-line types (i.e., NA19119-lymphoblastoid, WM2664-melanoma, MDA-MB-436-breast carcinoma, and HEK-293-kidney cell lines). Cells (2.5x10^-5^/100μl) were seeded into 96-well plates and treated with the maximum concentration of drug in duplicate for 12 hours. A total of 30μM DCFDA (AdipoGen Life Sciences) (or 5μM CellROX Green in later experiments) was then added to the wells and fluorescence was measured at 485ex/535em at 1 h using a Tecan Infinite 200 plate reader (Tecan Group Ltd.).

#### mRNA expression assays

LCLs were identified as sensitive or resistant based on their mean AUC value across the four drug treatments: arsenic trioxide, erlotinib, trametinib, and the paclitaxel + epirubicin combination, which were flagged by our GWAS. Six cell lines each were used in the sensitive and resistant group. Total RNA was isolated from ~1x10^-6^ cells using the RNeasy kit (Qiagen) and then reverse-transcribed using the Verso cDNA kit (ThermoFisher Scientific). RT-PCR reactions were subsequently run in triplicate on a QuantStudio 6 Flex Real-Time PCR System (ThermoFisher Scientific) using PowerUp Sybr Green Master Mix (ThermoFisher Scientific). Primers used included human *NQO1* (forward: GGTTTGGAGTCCCTGCCATT, reverse: GCCTTCTTACTCCGGAAGGG); *NFAT5* (forward: GTCAGTGGGAATATATGTAGTG, reverse: GTTTTCATTGCTTTCATGGC); and GAPDH (forward: CTTTTGCTGCGCCAG, reverse: TTGATGGCAACAATATCCAC). Reactions were conducted in 10-μl volumes using 500 nmol of each primer. Thermocycler parameters were: 50°C for 2 min, 95°C for 10 min, and then 40 cycles of 95°C for 15 s followed by 60°C for 1 min. The data were analyzed using the delta C_t_ method normalized against GAPDH. Each cell sample was measured in triplicate. The means (± standard deviation) for each cell line were averaged for each group.

#### *NQO1* enzyme activity assays

*NQO1* enzymatic activity was measured using the *NQO1* Activity Assay Kit (ab184867) from Abcam (Cambridge, UK). Briefly, the *NQO1* activity assay is based on the dicoumarol-sensitive reduction of WST-1 in the presence of menadione using 10 ug of cellular lysate protein in a 96-well plate format. Progress of the reaction was measured at 1-min intervals by measuring absorbance at 450 nm on an Infinite F200 microplate reader (Tecan Group Ltd). A 10-min endpoint reading was chosen as a time point within the linear region of the reaction. Replicates were averaged, and activity was expressed as the OD_450nm_ following subtraction of OD_450nm_ + dicoumarol.

#### *NQO1* GFP reporter plasmid cloning

The *NQO1* promoter-reporter plasmid was constructed using pTRF.1 udsVenus (a gift from Kevin Janes, Addgene plasmid # 58692; http://n2t.net/addgene:58692; RRID:Addgene_58692). The JunD promoter was removed by restriction digestion with EcoRI and SpeI, followed by gel purification. The *NQO1* promoter inserts were prepared by PCR of gDNA from either a cell line that was homozygous wildtype for rs689457 (GM19201) or a homozygous variant (GM01359) using primers AAAAAGAATTCTAGACCCACCTCGGCCTCCCATATTGC and AAAAACTAGTTATCCTGTCCGGCCCGTTTGAGG containing EcoRI and SpeI sites in the 5’ ends, respectively. PCR was followed by restriction digestion before ligation into the pTRF.1udsVenus plasmid backbone. The rs689457 SNP was confirmed by sequencing in the resulting plasmids, pNQO1-wt-GFP and pNQO1-var-GFP.

#### *NQO1* knockdown assays

Stable *NQO1* knockdown cell lines were prepared by transduction with lentivirus-encoding *NQO1*-specific shRNA sequences (Open Biosystems TRC1 library) obtained through the Lenti-shRNA Core Facility (University of North Carolina). Corresponding control cell lines were prepared using a scrambled shRNA sequence. To obtain virus, low-passage HEK293T cells were transfected with pLKO.1 plasmids encoding five individual *NQO1* shRNAs, the packaging plasmid pMDG.2, and the envelope plasmid pCMV-VSV-G at a ratio of 1:0.75:0.25 with Transporter5 transfection reagent (Polysciences Inc., Warrington, PA, USA). The media was replaced with fresh media 18 h after transfection, and viral particles were collected twice at 24-h intervals thereafter. Viral supernatants from the five transfections and two collection times were then pooled and filtered through a 0.45-μm cellulose acetate filter. Virus was concentrated ~50-fold by adding PEG-8000 and NaCl to a final concentration of 40% (w/v) and 1.2M, respectively, in PBS at pH7.0 and then storing overnight at 4°C. Virus was then centrifuged at 1600xg for 45 min, and the pellet was resuspended in cell culture media. Cells were subsequently transduced with viral supernatant supplemented with polybrene (4 μg/ml; Sigma-Aldrich, St. Louis, MO, USA). After 48 h, positive selection for transduced cells was conducted using 1 μg/ml puromycin for 10 days. The level of knockdown was determined by western blotting performed using a mouse anti-*NQO1* antibody (Thermo Scientific, Waltham, MA, USA). Anti-GAPDH (Proteintech, Rosemont, IL, USA) was used as a load control. Anti-fluor-conjugated secondary antibodies from LiCOR were used as needed, and detection was carried out on a LiCOR Odyssey to visualize immunoreactive bands (LI-COR Biosciences, Lincoln, NE, USA).

## Supporting information

S1 Text Fig A. Manhattan plots of MAGWAS p-values for the 44 drug treatments used in this study. Manhattan plots of MAGWAS -log_10_ (p-value) over 22 autosomes for the association of genotype and cell viability for the 44 drug treatments used in this study. The dashed and solid lines indicate the thresholds for the genome-wide suggestive significance level of 10^−6^ and the genome-wide significance level of 10^−8^, respectively. Drug treatments: - APA:Apatinib, ARSEN:Arsen, AXI:Axitinib, AZA:Azacytidine, CAB:Cabozantinib, CARBO:Carboplatin, CLAD:Cladaribine, CRIZ:Crizotinib, CYTAR:Cytosine beta d’arabinoside, DAS:Dasatinib, DAUN:Daunorubicin, DOC:Docetaxel, DOV:Dovitinib, DOX:Doxorubicin, EPI:Epirubicin, ERL:Erlotinib, ETOP:Etoposide, FLOX:Fluoro-deoxyuridine, FLUD:Fludarabine, 5FU:5-Fluorouracil, GEM:Gemcitabine, HYDROX:Hydroxyurea, IBRU:Ibrutinib, IDA:Idarubicin, MAS:Masatinib, MIT:MitomycinC, MOX:Mitoxantrone, NIL:Nilotinib, NIN:Nintedanib, OXAL:Oxaliplatin, PAC:Paclitaxel, SOR:Sorafenib, SUN:Sunitinib, TEMO:Temozolomide, TENI:Teniposide, TIV:Tivantinib, TOPO:Topotecan, TRA:Trametinib, VAN:Vandetanib, VEM:Vemurafenib, VINB:Vinblastine, VINC:Vincristine sulfate, VINO:Vinorelbine, SYN:Paclitaxel+Epirubicin combination treatment. **Fig B. LocusZoom plots of the genes surrounding SNP rs1800566 on chromosome 16.** LocusZoom plots showing the regional genes surrounding a 1 mega base pair region around SNP rs1800566 on chromosome 16 for associations with the drug treatments (A) arsenic trioxide, (B) paclitaxel + epirubicin, and (C) trametinib. Multiple SNPs are used as lead/reference SNPs (shown as diamonds). For all other non-lead SNPs (shown as circles and triangles), their color and shape are matched to the lead SNP with which it is in the highest linkage disequilibrium (LD), as shown in the legend. The extent of LD with the lead SNP is shown by the color gradient. **Fig C. Regional genes plot of chromosome 16 near the *NFAT5* and *NQO1* genes for erlotinib from the genome-wide association analysis after controlling for SNP rs1800566.** A LocusZoom plot showing the regional genes surrounding a 1 mega base pair region near the *NFAT5* and *NQO1* genes on chromosome 16 for associations with the drug erlotinib, after controlling for the effects of SNP rs1800566 in the *NQO1* gene. The peak previously seen in this region, shown in Fig 2, is absent, and no SNPs in this genomic region were significantly associated with drug response in this controlled analysis, indicating that SNP rs1800566 is almost exclusively responsible for the association signal in our study and is likely the functional SNP. The extent of LD with the lowest p-value SNP, rs12447326, is shown by the color gradient. **Fig D. Dose-response profiles for trametinib stratified by genotype at SNP rs1800566.** C is the reference allele, and T is the variant allele at SNP rs1800566. At lower concentrations, individuals with the CC genotype have lower cell viability than others, while at higher concentrations, they have higher cell viability than others. Individuals with the CT genotype have intermediate cell viability compared to CC and TT individuals. The numbers of individuals for each genotype are: CC—349, CT—283, and TT—48. Concentrations are on the log10 scale on the X-axis. The bars represent the standard error of the mean. **Fig E. Dose-response profiles for paclitaxel + epirubicin combination treatment stratified by genotype at SNP rs1800566.** C is the reference allele, and T is the variant allele at SNP rs1800566. The numbers of individuals for each genotype are: CC—349, CT—283, and TT—48. Concentrations are on the log10 scale on the X-axis. The bars represent the standard error of the mean. **Fig F. Quantile-quantile plots of MAGWAS p-values.** Quantile-quantile plots showing the deviation of the observed MAGWAS -log_10_ (p-values) from the null hypothesis for the drug treatments: (A) arsenic trioxide, (B) erlotinib, (C) paclitaxel + epirubicin, and (D) trametinib. **Fig G. Drug-induced gene expression and enzymatic activity of NQO1.** The genotype at SNP rs689457 influences both *NQO1* mRNA expression and *NQO1* enzymatic activity in LCLs. (A) *NQO1* protein activity measured using the *NQO1* Activity Assay Kit (ab184867) from Abcam (Cambridge, UK) in three homozygous reference and three homozygous variant LCLs treated with the GWAS-flagged compounds at the half-maximal concentration used in the study at a 10-min endpoint. (B) *NQO1* mRNA expression measured by qPCR in three homozygous reference and three homozygous variant LCLs treated with the GWAS-flagged compounds at the half-maximal concentration used in the study following 24 h treatment. (C) qPCR of *NQO1* with an *NQO1* promoter GFP reporter plasmid transiently transfected into the HEK-293 cell lines treated with the GWAS-flagged compounds at the half-maximal concentration used in this study following 24 h treatment. The bars show the mean of the cell lines per genotype, and the vertical lines represent the standard error of the mean. Drug treatments: Ars: Arsenic, Erl: Erlotinib, Tra: Trametinib, Syn: Paclitaxel+Epirubicin combination treatment. Statistical significance symbols: ns: p > 0.05, *: p < = 0.05, **: p < = 0.01, ***: p < = 0.001, ****: p < = 0.0001. **Fig H. Drug-induced gene expression of NFAT5 target genes–S100A4 and SMIT.** Minimal drug-induced transcriptional activation of *NFAT5* measured by expression of its target genes was observed. Transcriptional activity of *NFAT5* target genes (A) *S100A4* and (B) *SMIT* was measured by qPCR following 24 h treatment of an LCL (NA19119) with GWAS-flagged compounds (red bars) as well as compounds representative of the various drug classes (grey bars) at the maximum concentration used in the cell viability screening in this study. NaCl (90mM) was included as a positive control. Drug treatments: Ars: Arsenic, Epi: Epirubicin, Erl: Erlotinib, Gem: Gemcitabine, Hydrox: Hydroxyurea, Mit: MitomycinC, Pac: Paclitaxel, Tra: Trametinib, NaCl: Sodium Chloride. Statistical significance symbols: ns: p > 0.05, *: p < = 0.05, **: p < = 0.01, ***: p < = 0.001, ****: p < = 0.0001. **Fig I. Drug-induced cell viability and cellular ROS assays in *NQO1* knockdown cells.** Knockdown of *NQO1* resulted in increased reactive oxygen species (ROS) and increased sensitivity to several drug treatments used in this study. We measured cell viability using the alamarBlue assay in empty vector and *NQO1* knockdown. (A) LCL NA19119, (B) kidney cell line HEK-293, and (C) melanoma cell line WM2664 after a 48h treatment at half-maximal concentration in the cell viability screening of GWAS-flagged compounds (red/pink bars) as well as compounds representative of the various drug classes in this study (dark grey/light grey bars). We measured ROS production using a DCFDA cellular ROS assay kit in empty vector and *NQO1* knockdown. (D) LCL NA19119, (E) kidney cell line HEK-293, and (F) melanoma cell line WM2664 after an 18h treatment at the half-maximal concentration used in the viability screening of GWAS-flagged compounds (red/pink bars) as well as compounds representative of the various drug classes in this study (dark grey/light grey bars). Drug treatments: 5FU: 5-Fluorouracil, Ars: Arsen, Epi: Epirubicin, Erl: Erlotinib, Hydrox: Hydroxyurea, Mit: MitomycinC, Syn: Paclitaxel+Epirubicin combination treatment, Teni: Teniposide, Tra: Trametinib, Vinc: Vincristine. Statistical significance symbols: ns: p > 0.05, *: p < = 0.05, **: p < = 0.01, ***: p < = 0.001, ****: p < = 0.0001. **Table A. Anticancer drug treatments and their concentrations used for the drug-response assays.** The 44 anticancer drug treatments and their six associated concentrations used for the drug-response assays in LCLs in this study. Concentrations are in mM. **Table B. SNPs significantly associated with drug response from MAGWAS after controlling for SNP rs1800566.** SNPs associated with the multivariate response for each drug at the genome-wide suggestive significance level or higher from the genome-wide association using MAGWAS when controlling for the effects of SNP rs1800566 in the *NQO1* gene. The results are sorted by the host gene and drug. The SNPs that were not suggestively significant in the original genome-wide association mapping reported in Table 2 are shown in **bold**. Chr: Chromosome, The most severe consequences were obtained from Ensembl VEP (Ensembl release 97 –July 2019). **Table C. Significant results from multivariate linear regression of drug response on the baseline expression of *NQO1* and *NFAT5* transcripts.** Significant correlations between drug response and baseline expression of *NQO1* and *NFAT5* transcripts after multiple testing correction with a false discovery rate of q < 0.25 applied per drug. **Table D. p-values for rs1800566 at each stage of the pQTL model.** The Stage 1 column shows the p-values for the association of rs1800566 with the AUC for each drug treatment. The Stage 2 column shows the p-value for the association of rs1800566 with NQO1 protein activity. The Stage 3 column shows the p-values for the association of NQO1 protein activity with the AUC for each drug treatment. ‘*’ indicates statistical significance at p-value < 0.05. **Table E. Estimates and p-values for the covariate ‘NQO1_protein_activity’ from Stage 3 of the pQTL model for the linear regression of AUC on the NQO1 protein activity stratified by genotype at SNP rs1800566.** CC = reference genotype, CT = heterozygous genotype, TT = homozygous alternate genotype.
(DOCX)
