# Supplementary material for: High-throughput screening and genome-wide analyses of 44 anticancer drugs in the 1000 Genomes cell lines reveals an association of the NQO1 gene with the response of multiple anticancer drugs
Source: PLoS Genet. 2021 Aug 26;17(8):e1009732. doi: 10.1371/journal.pgen.1009732 (PMC8439493; doi:10.1371/journal.pgen.1009732)
Supplement: S1 Text — Manhattan plots of MAGWAS -log10 (p-value) over 22 autosomes for the association of genotype and cell viability for the 44 drug treatments used in this study. The dashed and solid lines indicate the thresholds for the genome-wide suggestive significance level of 10−6 and the genome-wide significance level of 10−8, respectively. Drug treatments: - APA:Apatinib, ARSEN:Arsen, AXI:Axitinib, AZA:Azacytidine, CAB:Cabozantinib, CARBO:Carboplatin, CLAD:Cladaribine, CRIZ:Crizotinib, CYTAR:Cytosine beta d’arabinoside, DAS:Dasatinib, DAUN:Daunorubicin, DOC:Docetaxel, DOV:Dovitinib, DOX:Doxorubicin, EPI:Epirubicin, ERL:Erlotinib, ETOP:Etoposide, FLOX:Fluoro-deoxyuridine, FLUD:Fludarabine, 5FU:5-Fluorouracil, GEM:Gemcitabine, HYDROX:Hydroxyurea, IBRU:Ibrutinib, IDA:Idarubicin, MAS:Masatinib, MIT:MitomycinC, MOX:Mitoxantrone, NIL:Nilotinib, NIN:Nintedanib, OXAL:Oxaliplatin, PAC:Paclitaxel, SOR:Sorafenib, SUN:Sunitinib, TEMO:Temozolomide, TENI:Teniposide, TIV:Tivantinib, TOPO:Topotecan, TRA:Trametinib, VAN:Vandetanib, VEM:Vemurafenib, VINB:Vinblastine, VINC:Vincristine sulfate, VINO:Vinorelbine, SYN:Paclitaxel+Epirubicin combination treatment. Fig B. LocusZoom plots of the genes surrounding SNP rs1800566 on chromosome 16. LocusZoom plots showing the regional genes surrounding a 1 mega base pair region around SNP rs1800566 on chromosome 16 for associations with the drug treatments (A) arsenic trioxide, (B) paclitaxel + epirubicin, and (C) trametinib. Multiple SNPs are used as lead/reference SNPs (shown as diamonds). For all other non-lead SNPs (shown as circles and triangles), their color and shape are matched to the lead SNP with which it is in the highest linkage disequilibrium (LD), as shown in the legend. The extent of LD with the lead SNP is shown by the color gradient. Fig C. Regional genes plot of chromosome 16 near the NFAT5 and NQO1 genes for erlotinib from the genome-wide association analysis after controlling for SNP rs1800566. A LocusZoom plot showing the regional genes [file pgen.1009732.s001.docx]

## S1 Text

**MAGWAS Analysis**

The observed cell viability in lymphoblastoid cell lines (LCLs) in our assays for the following drugs did not show an average response correlated with increasing concentrations and hence we removed these drugs from any further analyses: apatinib, axitinib, azacytidine, cabozantinib, dasatinib, and nilotinib. We excluded single nucleotide polymorphisms (SNPs) with less than 20 individuals for any genotype from the association analysis to prevent violations of normality for the MANCOVA method. We used the Pillai-Bartlett trace to calculate p-values. A cut-off of p<1x10^-6^ was used to determine suggestive associations, and a cut-off of p<1x10^-8^ was used to determine statistically significant association [1]. Figure F shows the quantile-quantile plots for the p-values obtained from MAGWAS for the following drug treatments: arsenic trioxide, erlotinib, paclitaxel + epirubicin, and trametinib.

To identify other genome-wide variants that may be associated with response to the drugs in our assay, we repeated the multivariate GWAS while controlling for the SNP rs1800566 using the same model and covariates, with SNP rs1800566 as an additional covariate.

**Gene Expression Two-stage Regression**

For the regression analysis, the stages are specified as:

- First stage: G_i_ = β_0_ + **L**_i_***β** + ϵ_i_  , ϵ_i_ ~ N(0, σ^2^)
- Second stage: 𝒀_𝒊𝒋_ = 𝛽_0_ + 𝛽_1_*𝑃𝐶1_𝑖_ + 𝛽_2_*𝑃𝐶2_𝑖_ + 𝛽_3_*𝑃𝐶3_𝑖_ + 𝛽_4_*𝑆𝑒𝑥_𝑖_ + 𝛽_5_*ϵ_i_ + 𝒆_𝒊𝒋_ , 𝒆**_𝑖𝑗_** ∼ 𝑁(𝟎,𝚺)

In the first stage, we performed linear regression using the limma package [2] to remove the lab batch effects, where G_i_ is the quality-controlled RNA-Seq read count of gene *g* for individual *i*, L_i_ is a vector of indicator variables for the lab in which the RNA-Seq was conducted for individual *i*, β is a vector of the regression parameters, and ϵ_i_ are the residuals = observed read count for gene *g* - predicted gene read count for gene for individual *i*. To prevent extreme residual values from having high leverage on parameter estimates, we log-transformed and standardized residuals before using them in the second stage. In the second stage, we performed linear regression using the R function *lm()* in package *stats v3.4.0* [3] for each gene *g*, where Y_ij_ is the vector of normalized responses for the six concentrations of the drug for individual *i*; PC_1_, PC_2_, and PC_3_ are the Eigenvalues from the first three principal components calculated using EigenStrat [4]; and sex is an indicator variable denoting the sex of individual *i*. We obtained significant results from the second stage after correcting for multiple testing on a per-drug basis using the Bonferroni correction with a significance level of p<0.05 for the gene expression analysis and the Benjamini–Hochberg method [5] with a false discovery rate of q<0.25 for the transcript expression analysis (R function *p.adjust()*, package *stats v3.4.0*) [3].

**Protein QTL Analysis**

For each stage, we used a linear regression model specified as follows:

- Stage 1: AUC_𝒊_ = 𝛽_0_ + 𝛽_1_*𝑃𝐶1_𝑖_ + 𝛽_2_*𝑃𝐶2_𝑖_ + 𝛽_3_*𝑃𝐶3_𝑖_ + 𝛽_4_*𝑆𝑒𝑥_𝑖_ + 𝛽_5_* rs1800566_i_ + e_ij_ , e_ij_ ∼ 𝑁(0, σ^2^)
- Stage 2: NQO1_protein_activity_i_ = 𝛽_0_ + 𝛽_1_*𝑆𝑒𝑥_𝑖_ + 𝛽_2_*rs1800566_i_ + e_ij_ , e_ij_ ∼ 𝑁(0, σ^2^)
- Stage 3: AUC_𝒊_ = 𝛽_0_ + 𝛽_1_*𝑃𝐶1_𝑖_ + 𝛽_2_*𝑃𝐶2_𝑖_ + 𝛽_3_*𝑃𝐶3_𝑖_ + 𝛽_4_*𝑆𝑒𝑥_𝑖_ + 𝛽_5_*NQO1_protein_activity_i_ + e_ij_ , e_ij_ ∼ 𝑁(0, σ^2^)

where AUC is the area under the curve; PC1, PC2, and PC3 are the Eigenvalues from the first three principal components calculated using EigenStrat [4]; sex is an indicator variable denoting the sex of individual *i*; rs1800566 is the number of minor alleles at that SNP; and NQO1_protein_activity is the baseline *NQO1* protein activity measured for each cell line using the NQO1 Activity Assay Kit (ab184867) from Abcam (Cambridge, UK).

In the first stage, we performed linear regression to determine the association of rs1800566 with the AUC for the four drug treatments. We computed the AUC for each drug using the drug treatment responses at the six assayed concentrations. Due to the smaller sample size of 72 cell lines, we used a univariate measure of AUC instead of the entire multivariate response. We used the Eigenvalues from the first three principal components and sex as covariates. In the second stage, we performed a linear regression of *NQO1* protein activity on the SNP rs1800566 to determine if rs1800566 is associated with differential *NQO1* protein activity. We performed stepwise linear regression with the Akaike information criterion (AIC) with the Eigenvalues from the first three principal components, sex, and rs1800566 as covariates. Model selection indicated sex and rs1800566 as significant variables. In Stage 3, we regressed the AUC for each drug treatment onto *NQO1* protein activity with the Eigenvalues from the first three principal components and sex as the covariates. We used a significance cut-off of p<0.05 at each stage of the pQTL analysis for each drug. All linear regression models were fit using the R function *lm()* in package *stats v3.4.0* [3].


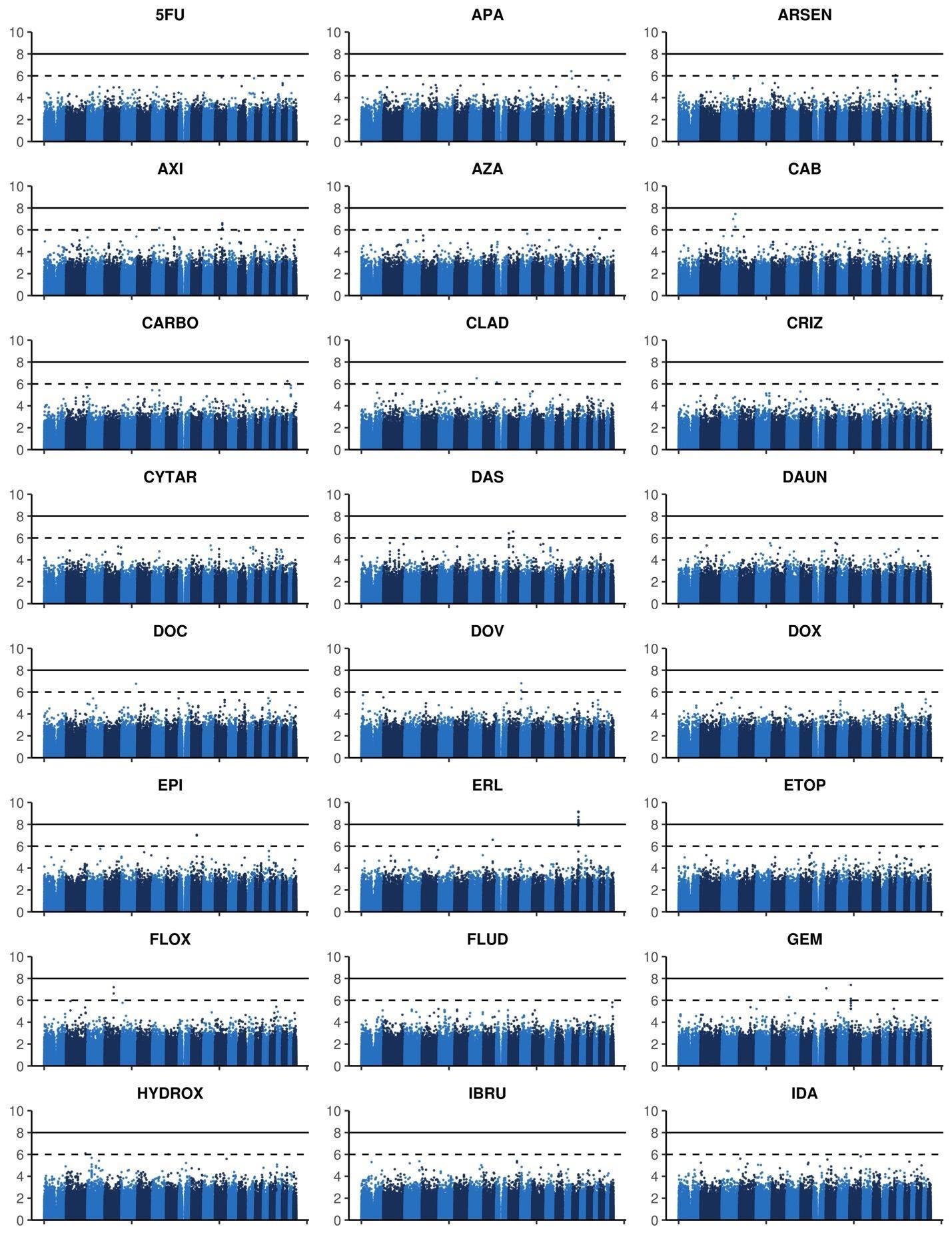


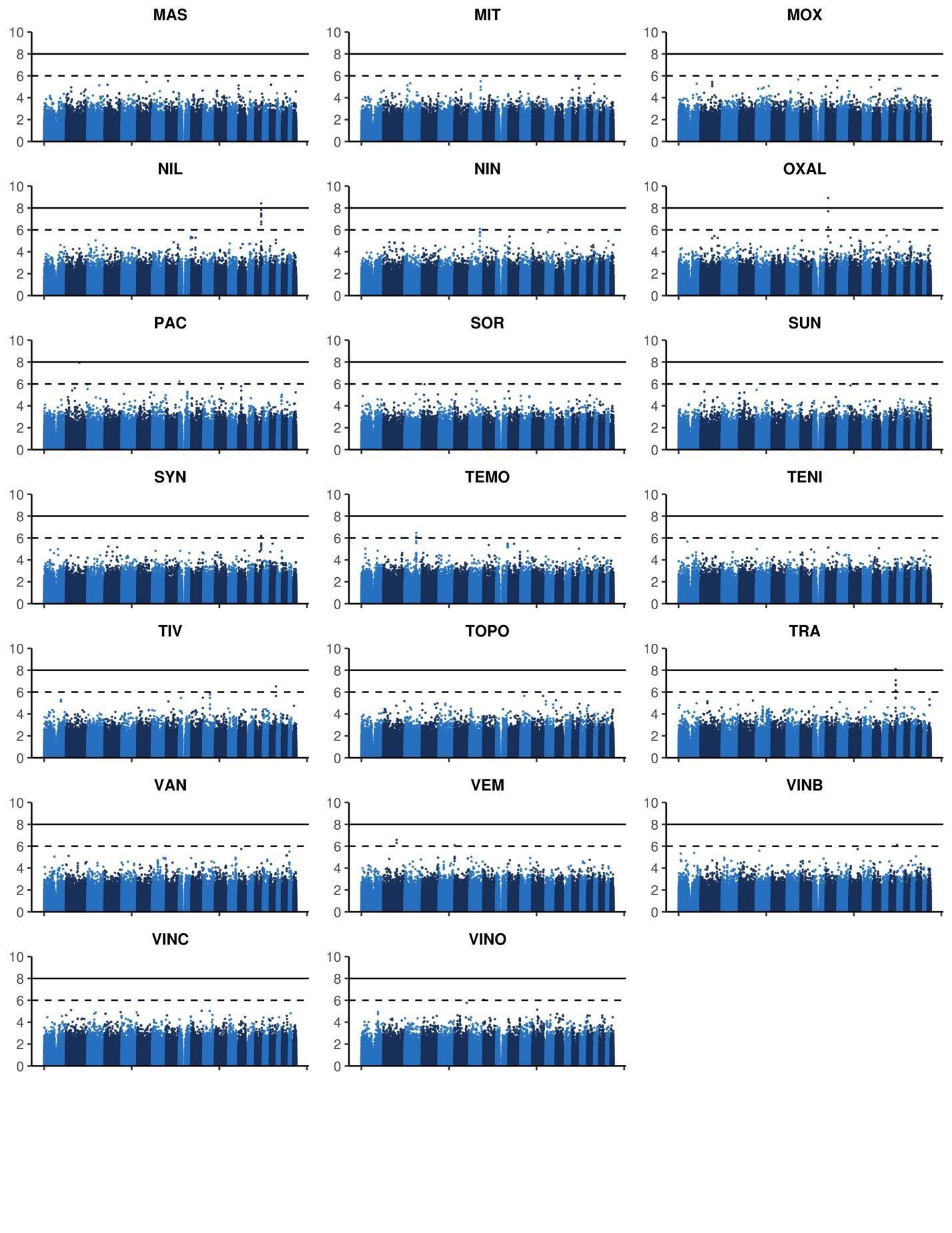


**Fig A. Manhattan plots of MAGWAS p-values for the 44 drug treatments used in this study.**

Manhattan plots of MAGWAS -log_10_ (p-value) over 22 autosomes for the association of genotype and cell viability for the 44 drug treatments used in this study. The dashed and solid lines indicate the thresholds for the genome-wide suggestive significance level of 10^-6^ and the genome-wide significance level of 10^-8^, respectively.

Drug treatments: APA:Apatinib, ARSEN:Arsen, AXI:Axitinib, AZA:Azacytidine, CAB:Cabozantinib, CARBO:Carboplatin, CLAD:Cladaribine, CRIZ:Crizotinib, CYTAR:Cytosine beta d’arabinoside, DAS:Dasatinib, DAUN:Daunorubicin, DOC:Docetaxel, DOV:Dovitinib, DOX:Doxorubicin, EPI:Epirubicin, ERL:Erlotinib, ETOP:Etoposide, FLOX:Fluoro-deoxyuridine, FLUD:Fludarabine, 5FU:5-Fluorouracil, GEM:Gemcitabine, HYDROX:Hydroxyurea, IBRU:Ibrutinib, IDA:Idarubicin, MAS:Masatinib, MIT:MitomycinC, MOX:Mitoxantrone, NIL:Nilotinib, NIN:Nintedanib, OXAL:Oxaliplatin, PAC:Paclitaxel, SOR:Sorafenib, SUN:Sunitinib, TEMO:Temozolomide, TENI:Teniposide, TIV:Tivantinib, TOPO:Topotecan, TRA:Trametinib, VAN:Vandetanib, VEM:Vemurafenib, VINB:Vinblastine, VINC:Vincristine sulfate, VINO:Vinorelbine, SYN:Paclitaxel+Epirubicin combination treatment

**(A)**

**
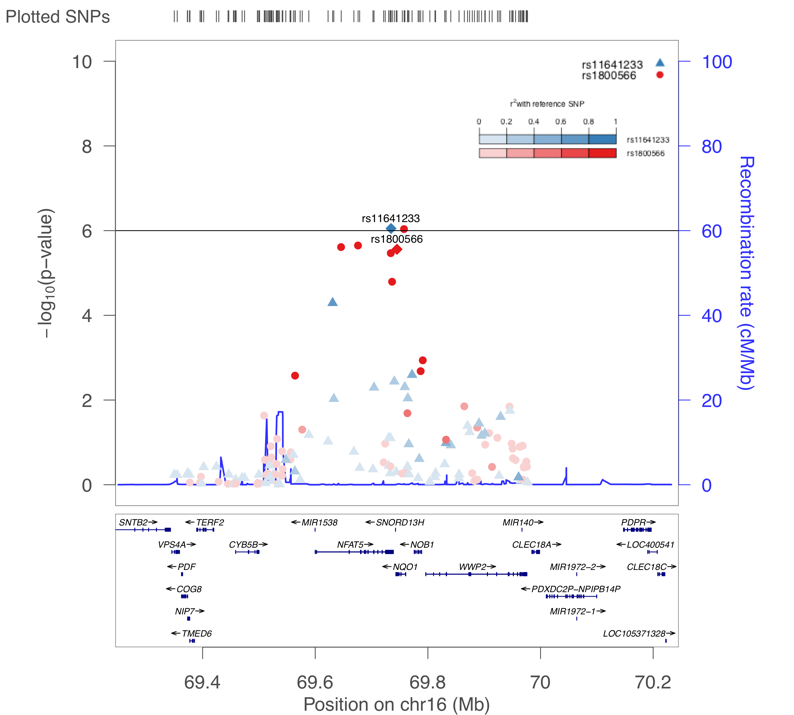
**

**(B)**

**
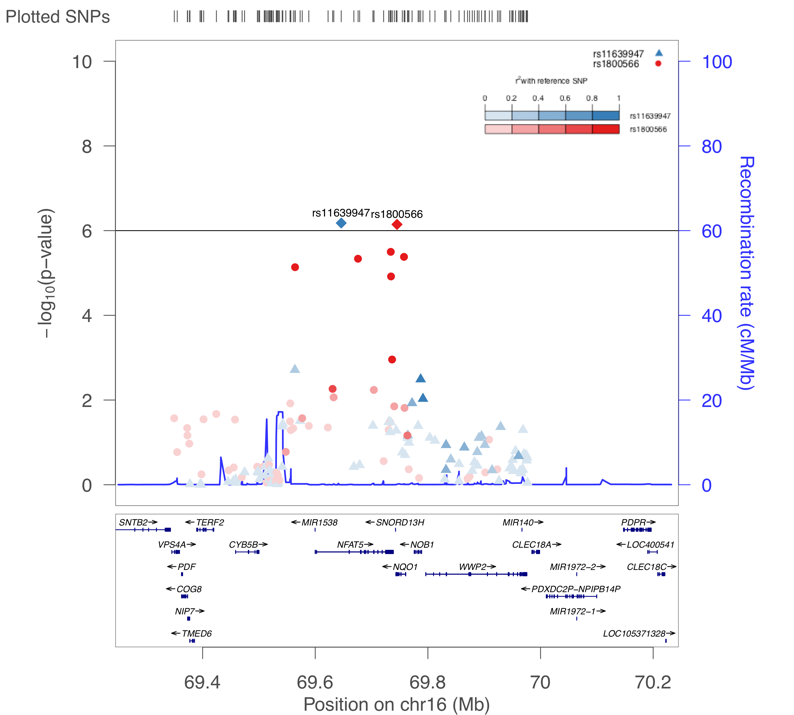
**

**(C)**

**
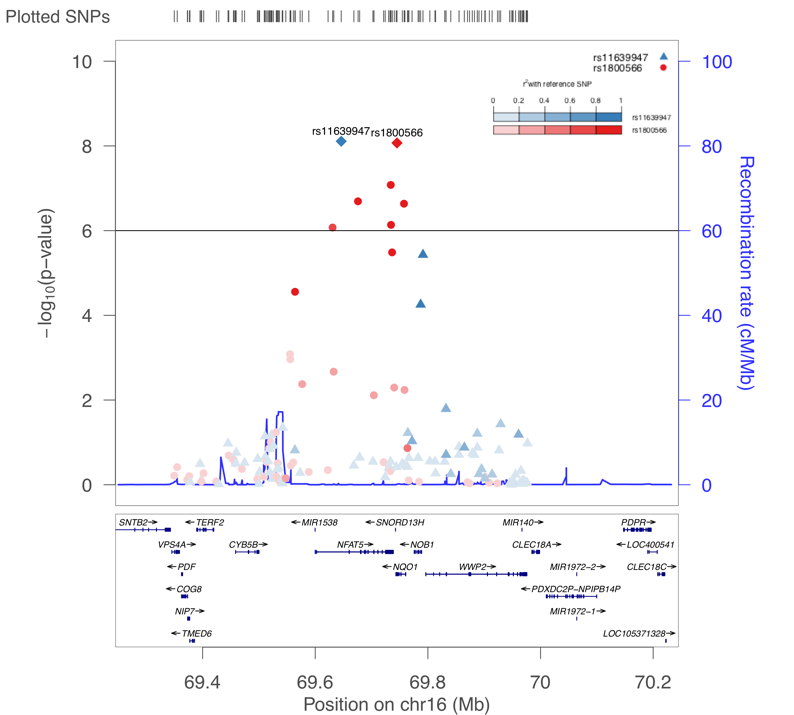
**

**Fig B. LocusZoom plots of the genes surrounding SNP rs1800566 on chromosome 16.**

LocusZoom plots showing the regional genes surrounding a 1 megabase pair region around SNP rs1800566 on chromosome 16 for associations with the drugs (A) arsenic trioxide, (B) paclitaxel + epirubicin, and (C) trametinib. Multiple SNPs are used as lead/reference SNPs (shown as diamonds). For all other non-lead SNPs (shown as circles and triangles), their color and shape are matched to the lead SNP with which it is in the highest linkage disequilibrium (LD), as shown in the legend. The extent of LD with the lead SNP is shown by the color gradient.


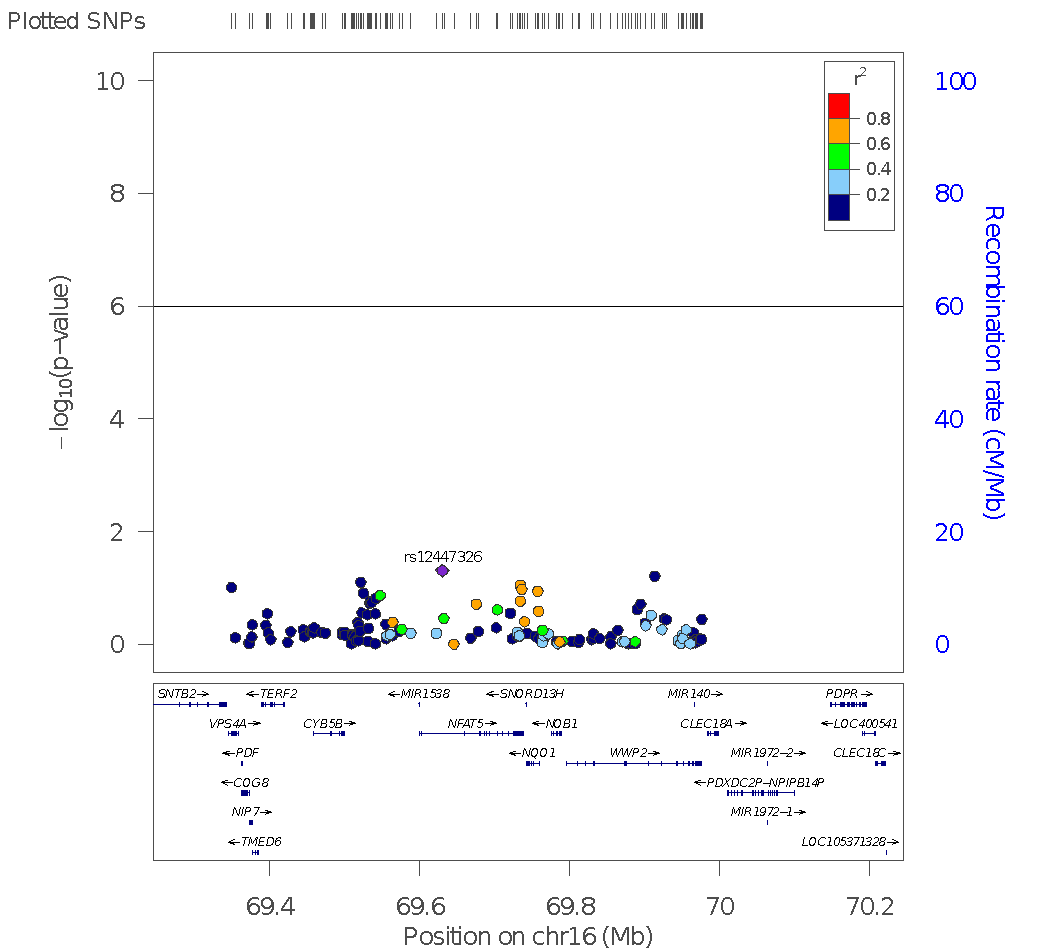


**Fig C. Regional genes plot of chromosome 16 near the *NFAT5* and *NQO1* genes for the drug erlotinib from the genome-wide association analysis after controlling for SNP rs1800566.**

A LocusZoom plot showing the regional genes surrounding a 1 megabase pair region near the *NFAT5* and *NQO1* genes on chromosome 16 for associations with the drug erlotinib, after controlling for the effects of SNP rs1800566 in the *NQO1* gene. The peak previously seen in this region, as shown in Fig 2, is absent, and no SNPs in this genomic region are significantly associated with drug response in this controlled analysis, indicating that SNP rs1800566 is almost exclusively responsible for the association signal in our study and is likely the functional SNP.

The extent of LD with the lowest p-value SNP, rs12447326, is shown by the color gradient.


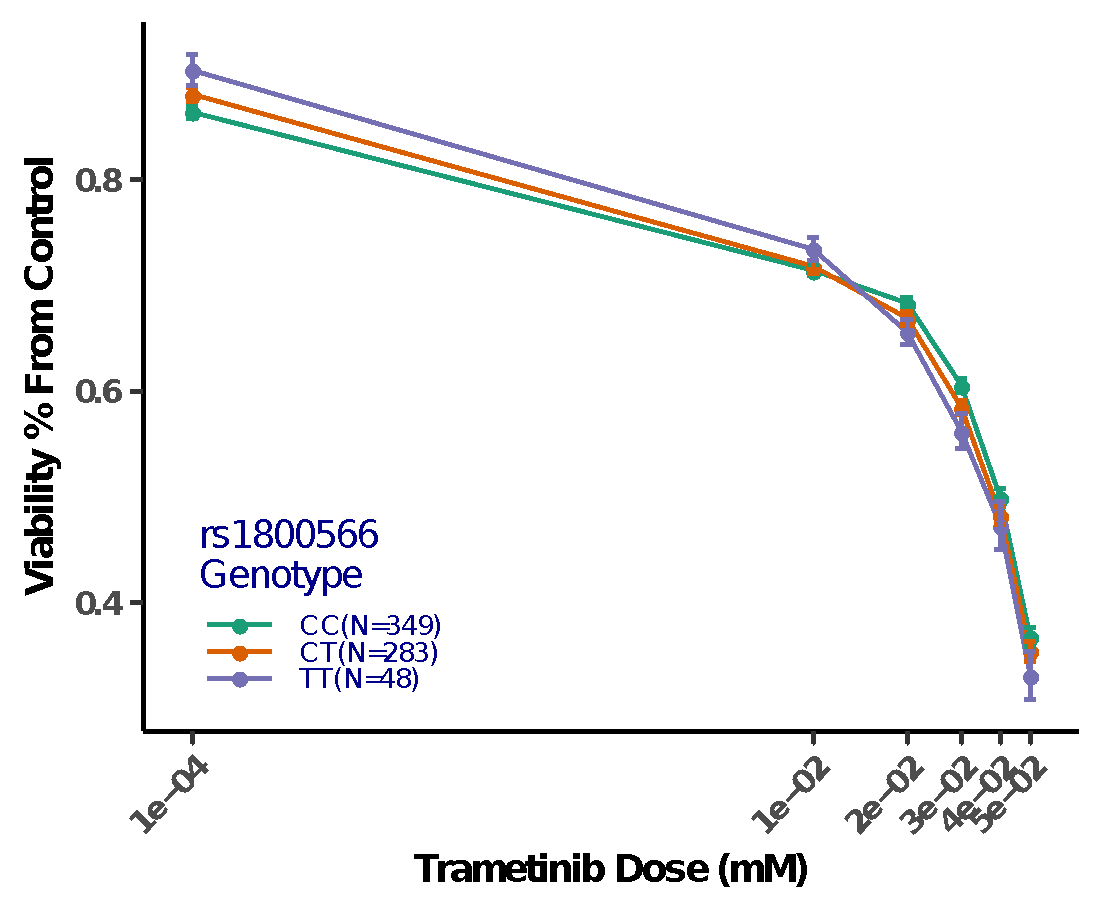


**Fig D. Dose-response profiles for trametinib stratified by genotype at SNP rs1800566.**

C is the reference allele, and T is the variant allele at SNP rs1800566. At lower concentrations, individuals with the CC genotype have lower cell viability than others, while at higher concentrations, they have higher cell viability than others. Individuals with the CT genotype have intermediate cell viability compared to CC and TT individuals. The numbers of individuals for each genotype are: CC - 349, CT - 283, and TT - 48. Concentrations are on the log10 scale on the X-axis. Bars represent the standard error of the mean.

**
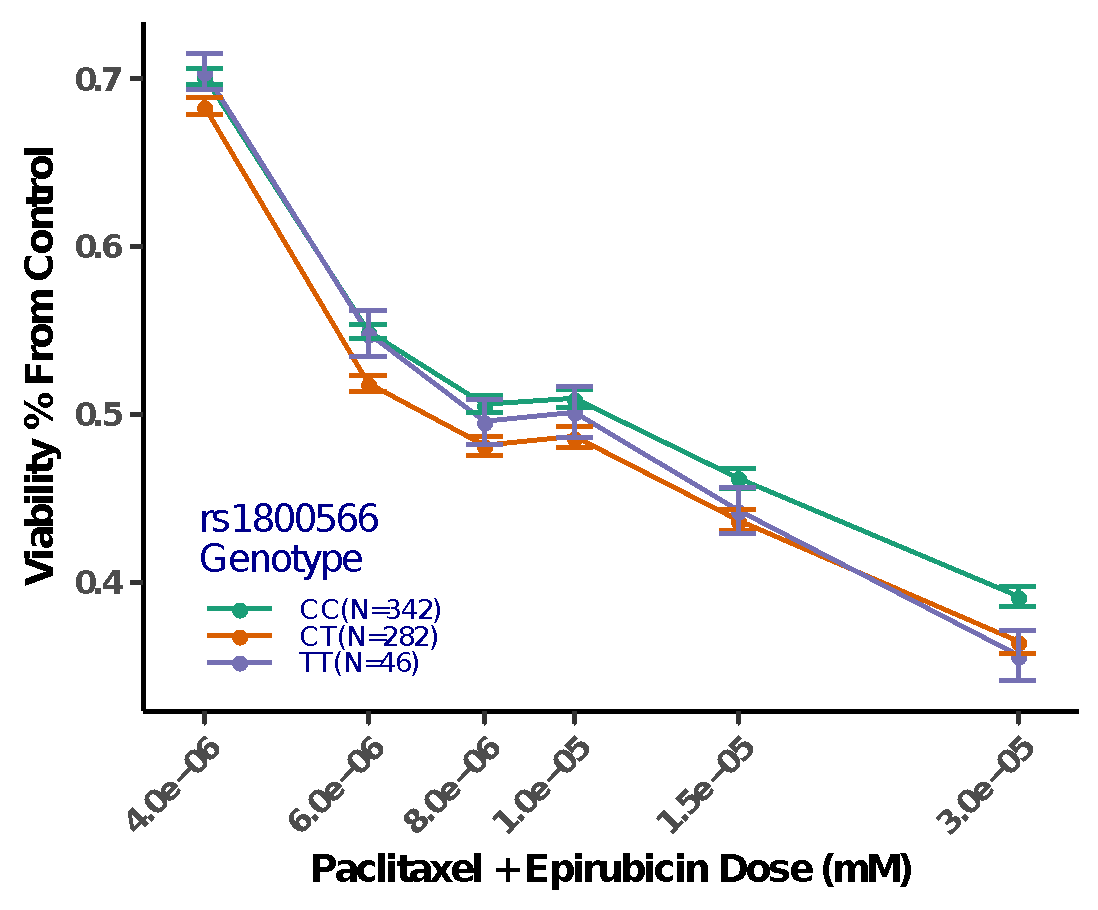
**

**Fig E. Dose-response profiles for paclitaxel + epirubicin combination treatment stratified by genotype at SNP rs1800566.**

C is the reference allele, and T is the variant allele at SNP rs1800566. The numbers of individuals for each genotype are: CC - 349, CT - 283, and TT - 48. Concentrations are on the log10 scale on the X-axis. Bars represent the standard error of the mean.

**(A) (B)**

**
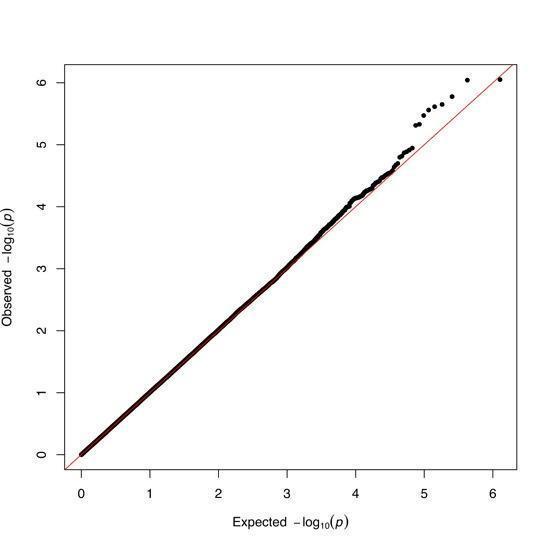
**

**(C)**

**
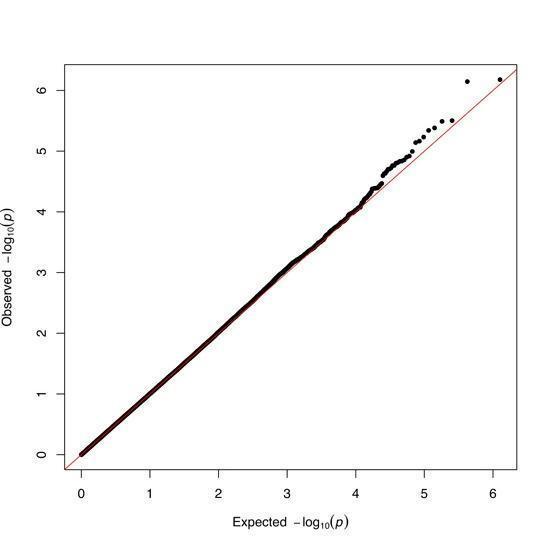
**

**
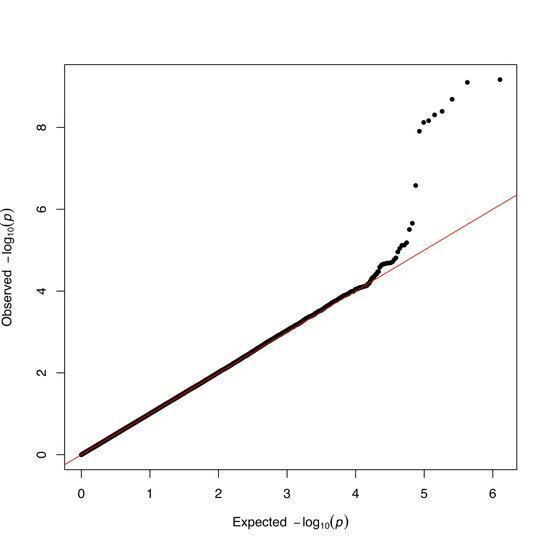
**

**(D)**

**
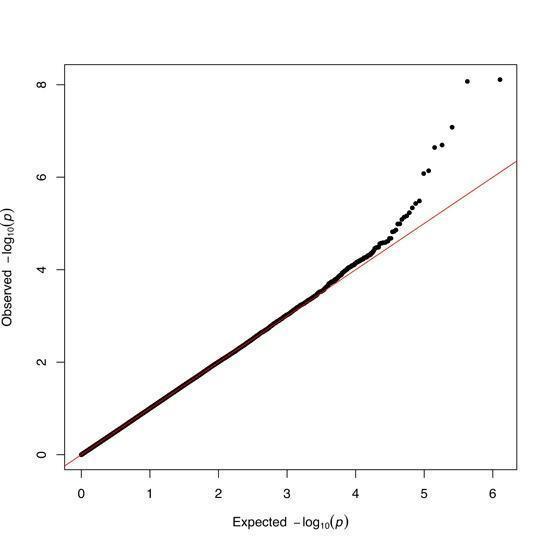
**

**Fig F. Quantile-quantile plots of MAGWAS p-values.**

Quantile-quantile plots showing the deviation of the observed MAGWAS -log_10_ (p-values) from the null hypothesis for the drug treatments: (A) arsenic trioxide (B) erlotinib (C) paclitaxel + epirubicin, and (D) trametinib.

**
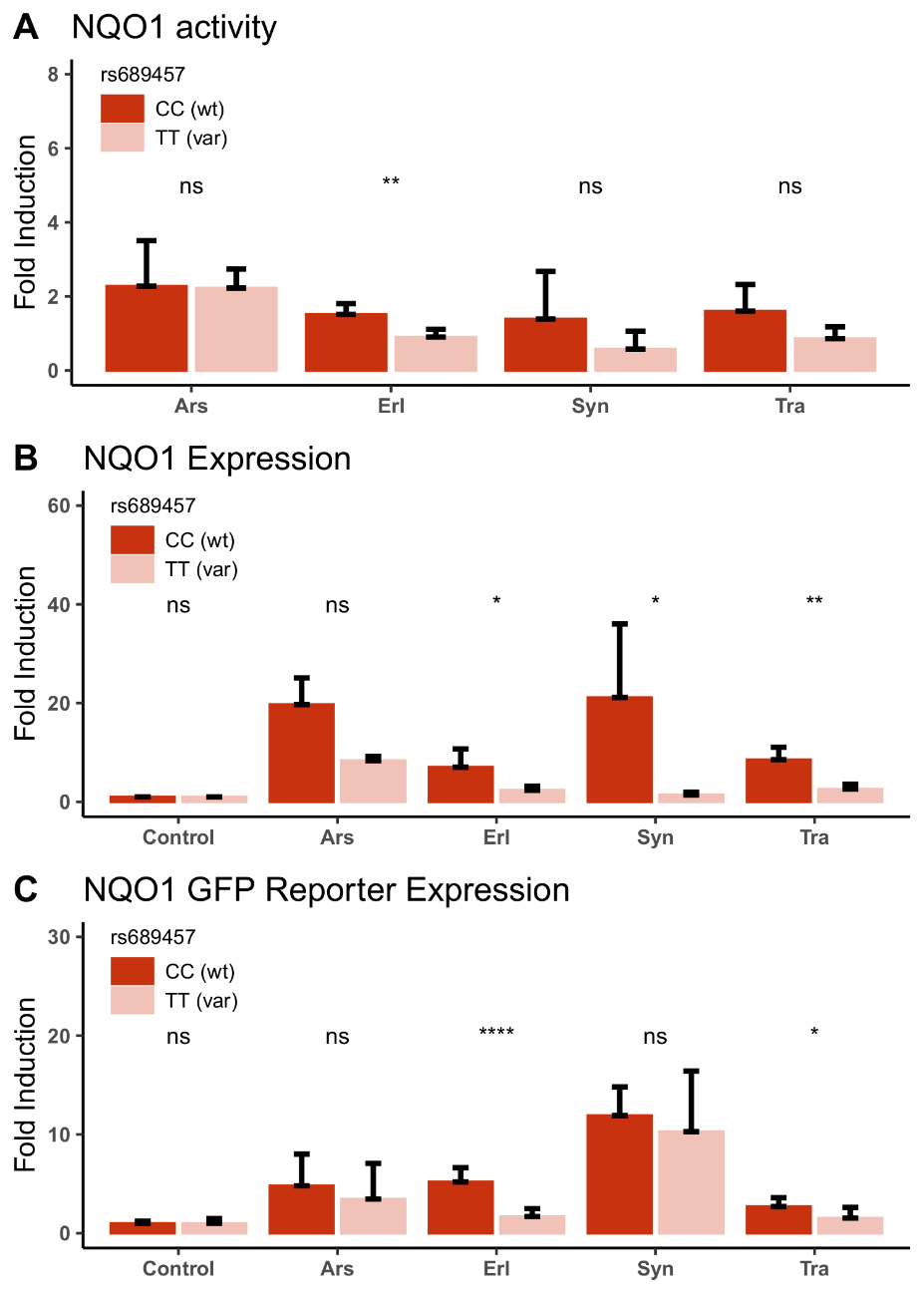
**

**Fig G. Drug-induced gene expression and enzymatic activity of NQO1.**

The genotype at SNP rs689457 influences both *NQO1* mRNA expression and *NQO1* enzymatic activity in LCLs. (A) *NQO1* protein activity measured using the NQO1 Activity Assay Kit (ab184867) from Abcam (Cambridge, UK) in three homozygous reference and three homozygous variant LCLs treated with the GWAS-flagged compounds at the half-maximal concentration used in the study at a 10-min endpoint. (B) *NQO1* mRNA expression measured using qPCR in three homozygous reference and three homozygous variant LCLs treated with the GWAS-flagged compounds at the half-maximal concentration used in the study following 24 h treatment. (C) qPCR of *NQO1* with an *NQO1* promoter GFP reporter plasmid transiently transfected into the HEK-293 cell lines treated with the GWAS-flagged compounds at the half-maximal concentration used in this study following 24 h treatment. The bars show the mean of the cell lines per genotype, and the vertical lines represent the standard error of the mean. Drug treatments: Ars: Arsen, Erl: Erlotinib, Tra: Trametinib, syn: Paclitaxel+Epirubicin combination treatment. Statistical significance symbols: ns: p>0.05, *: p<=0.05, **: p<=0.01, ***: p<=0.001, ****: p<=0.0001

**
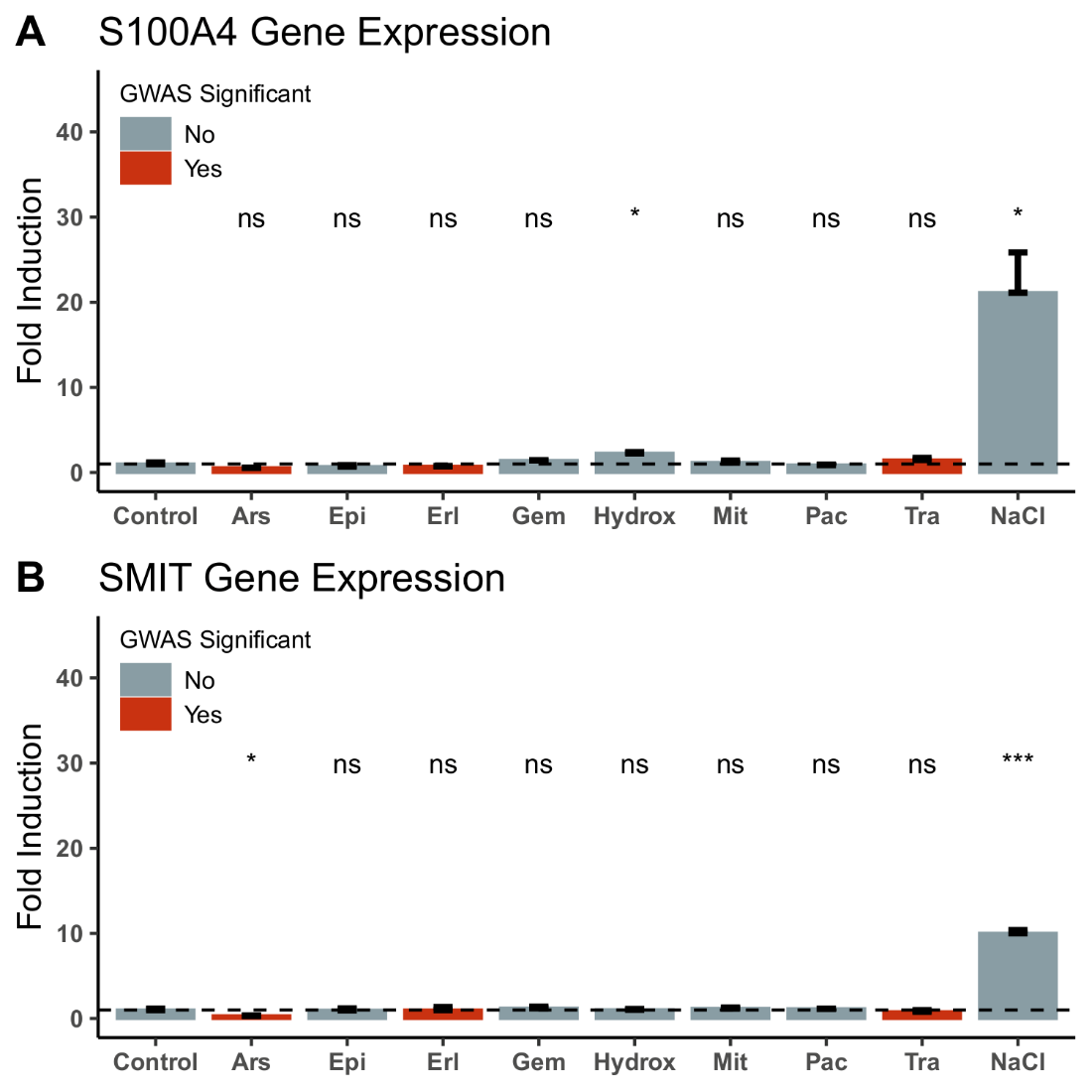
**

**Fig H. Drug-induced gene expression of *NFAT5* target genes – S100A4 and SMIT.**

We observed minimal drug-induced transcriptional activation of *NFAT5* measured by expression of its target genes. We measured transcriptional activity of *NFAT5* target genes (A) S100A4 and (B) SMIT using qPCR following 24 h treatment of an LCL (NA19119) with GWAS-flagged compounds (red bars) as well as compounds representative of the various drug classes (grey bars) at the maximum concentration used in the cell viability screening in this study. We included NaCl (90mM) as a positive control. Drug treatments: Ars: Arsen, Epi: Epirubicin, Erl: Erlotinib, Gem: Gemcitabine, Hydrox: Hydroxyurea, Mit: MitomycinC, Pac: Paclitaxel, Tra: Trametinib, NaCl: Sodium Chloride. Statistical significance symbols: ns: p>0.05, *: p<=0.05, **: p<=0.01, ***: p<=0.001, ****: p<=0.0001

**
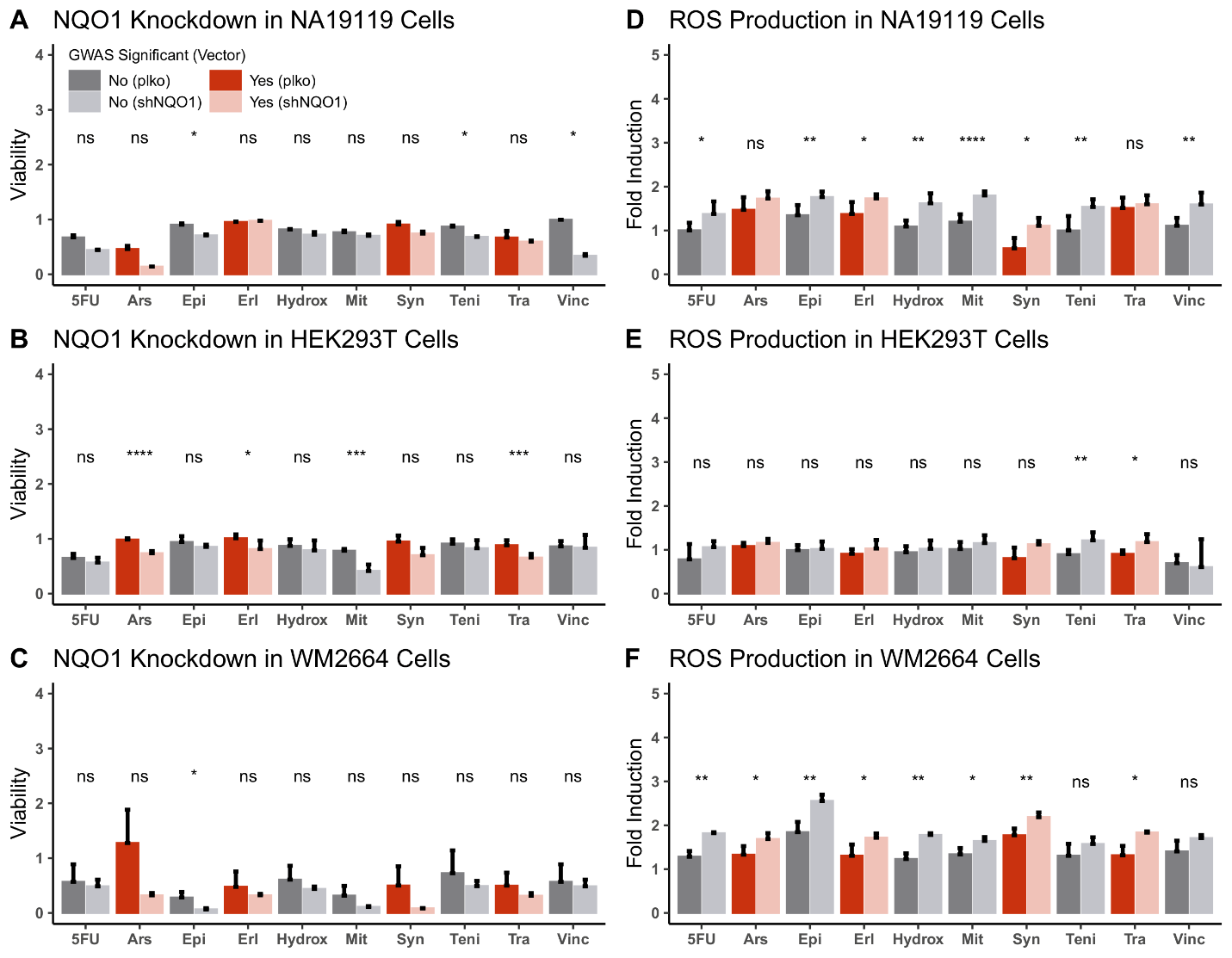
**

**Fig I. Drug-induced cell viability and cellular ROS assays in *NQO1* knockdown cells.**

Knockdown of *NQO1* resulted in increased reactive oxygen species (ROS) and increased sensitivity to several drug treatments used in this study. We measured cell viability using the alamarBlue assay in empty vector and *NQO1* knockdown. (A) LCL NA19119, (B) kidney cell line HEK-293, and (C) melanoma cell line WM2664 after a xxh treatment at xx concentration in the cell viability screening of GWAS-flagged compounds (red/pink bars) as well as compounds representative of the various drug classes in this study (dark grey/light grey bars). We measured ROS production using a DCFDA cellular ROS assay kit in empty vector and *NQO1* knockdown (D) LCL NA19119, (E) kidney cell line HEK-293, and (F) melanoma cell line WM2664 after a 12 h treatment at the maximum concentration used in the viability screening of GWAS-flagged compounds (red/pink bars) as well as compounds representative of the various drug classes in this study (dark grey/light grey bars). Drug treatments: 5FU: 5-Fluorouracil, Ars: Arsen, Epi: Epirubicin, Erl: Erlotinib, Hydrox: Hydroxyurea, Mit: MitomycinC, Syn: Paclitaxel+Epirubicin combination treatment, Teni: Teniposide, Tra: Trametinib, Vinc: Vincristine. Statistical significance symbols: ns: p>0.05, *: p<=0.05, **: p<=0.01, ***: p<=0.001, ****: p<=0.0001

**Table A. Anticancer drug treatments and their concentrations used for the drug-response assays.**

The 44 anticancer drug treatments and their six associated concentrations used for the drug-response assays in LCLs in this study. Concentrations are in mM.

|  | **Compound** | **Conc 1** | **Conc 2** | **Conc 3** | **Conc 4** | **Conc 5** | **Conc 6** |
| --- | --- | --- | --- | --- | --- | --- | --- |
| 1 | 5-Fluorouracil | 2.50E-01 | 1.00E-01 | 5.00E-02 | 2.00E-02 | 5.00E-03 | 1.00E-03 |
| 2 | Apatinib | 1.00E-01 | 5.00E-02 | 3.00E-02 | 2.50E-02 | 2.00E-02 | 1.00E-02 |
| 3 | Arsenic trioxide | 2.00E-02 | 1.50E-02 | 1.00E-02 | 8.00E-03 | 4.00E-03 | 3.00E-03 |
| 4 | Axitinib | 1.50E-02 | 1.00E-02 | 8.00E-03 | 6.00E-03 | 4.00E-03 | 2.00E-03 |
| 5 | Azacytidine | 1.20E-01 | 6.00E-02 | 4.00E-02 | 1.00E-02 | 2.50E-02 | 5.00E-03 |
| 6 | Cabozantinib | 1.00E-01 | 3.00E-02 | 1.00E-02 | 3.00E-03 | 1.00E-03 | 7.50E-04 |
| 7 | Carboplatin | 6.00E-02 | 4.00E-02 | 2.00E-02 | 1.00E-02 | 8.00E-03 | 4.00E-03 |
| 8 | Cladaribine | 2.00E-02 | 1.20E-02 | 8.00E-03 | 4.00E-03 | 2.00E-03 | 1.00E-03 |
| 9 | Crizotinib | 1.50E-02 | 1.00E-02 | 7.50E-03 | 5.00E-03 | 3.00E-03 | 2.50E-03 |
| 10 | Cytosine beta d arabinoside | 1.00E-02 | 5.00E-03 | 2.50E-03 | 1.40E-03 | 8.00E-04 | 4.00E-04 |
| 11 | Dasatinib | 9.00E-02 | 5.00E-02 | 5.00E-03 | 2.50E-03 | 1.00E-04 | 1.00E-05 |
| 12 | Daunorubicin | 8.00E-05 | 5.00E-05 | 2.50E-05 | 1.80E-05 | 1.00E-05 | 5.00E-06 |
| 13 | Docetaxel | 8.00E-03 | 5.00E-03 | 4.00E-03 | 3.00E-03 | 2.00E-03 | 1.00E-03 |
| 14 | Dovitinib | 1.50E-02 | 1.00E-02 | 7.50E-03 | 5.00E-03 | 2.00E-03 | 1.00E-03 |
| 15 | Doxorubicin | 2.50E-04 | 1.00E-04 | 7.50E-05 | 5.00E-05 | 1.30E-05 | 6.00E-06 |
| 16 | Epirubicin | 2.22E-04 | 1.00E-04 | 5.10E-05 | 2.80E-05 | 1.70E-05 | 6.40E-06 |
| 17 | Erlotinib | 5.00E-02 | 3.00E-02 | 2.00E-02 | 1.00E-02 | 8.00E-03 | 5.00E-03 |
| 18 | Etoposide | 2.50E-03 | 1.50E-03 | 7.50E-04 | 5.00E-04 | 2.50E-04 | 1.00E-04 |
| 19 | Fludarabine | 2.50E-01 | 1.50E-01 | 1.20E-01 | 6.70E-02 | 5.00E-02 | 2.50E-02 |
| 20 | Fluoro-deoxyuridine | 8.00E-01 | 1.00E-01 | 5.00E-02 | 2.00E-03 | 2.00E-04 | 2.00E-05 |
| 21 | Gemcitabine | 1.00E-04 | 4.00E-05 | 2.00E-05 | 9.00E-06 | 8.00E-06 | 5.00E-06 |
| 22 | Hydroxyurea | 4.00E-01 | 3.00E-01 | 2.00E-01 | 1.50E-01 | 7.50E-02 | 2.50E-02 |
| 23 | Ibrutinib | 5.00E-02 | 3.00E-02 | 1.00E-02 | 5.00E-03 | 1.00E-03 | 1.00E-04 |
| 24 | Idarubicin | 1.20E-04 | 8.00E-05 | 3.00E-05 | 2.00E-05 | 1.00E-05 | 5.00E-06 |
| 25 | Masatinib | 5.00E-02 | 3.00E-02 | 2.50E-02 | 2.00E-02 | 1.00E-02 | 5.00E-03 |
| 26 | MitomycinC | 1.00E-03 | 5.00E-04 | 3.00E-04 | 2.00E-04 | 8.00E-05 | 2.50E-05 |
| 27 | Mitoxantrone | 5.00E-04 | 2.50E-04 | 5.00E-05 | 1.00E-05 | 5.00E-06 | 5.00E-07 |
| 28 | Nilotinib | 1.20E-01 | 7.50E-02 | 5.00E-02 | 3.00E-02 | 2.00E-02 | 1.00E-02 |
| 29 | Nintedanib | 3.00E-02 | 1.00E-02 | 8.00E-03 | 5.00E-03 | 3.00E-03 | 1.00E-03 |
| 30 | Oxaliplatin | 2.00E-02 | 1.00E-02 | 5.00E-03 | 3.00E-03 | 1.50E-03 | 5.00E-04 |
| 31 | Paclitaxel | 3.00E-05 | 1.50E-05 | 1.00E-05 | 8.00E-06 | 6.00E-06 | 4.00E-06 |
| 32 | Paclitaxel + Epirubicin | 3.00E-05 | 1.50E-05 | 1.00E-05 | 8.00E-06 | 6.00E-06 | 4.00E-06 |
| 33 | Sorafenib | 3.00E-02 | 2.00E-02 | 1.50E-02 | 1.00E-02 | 8.00E-03 | 6.00E-03 |
| 34 | Sunitinib | 1.00E-02 | 8.00E-03 | 6.25E-03 | 4.20E-03 | 2.10E-03 | 1.00E-03 |
| 35 | Temozolomide | 1.50E+00 | 1.00E+00 | 7.50E-01 | 5.00E-01 | 2.50E-01 | 1.00E-01 |
| 36 | Teniposide | 2.00E-03 | 2.50E-04 | 1.00E-04 | 5.00E-05 | 1.00E-05 | 5.00E-06 |
| 37 | Tivantinib | 5.00E-02 | 1.00E-02 | 3.00E-03 | 7.50E-04 | 5.00E-04 | 7.50E-05 |
| 38 | Topotecan | 5.00E-05 | 2.50E-05 | 2.00E-05 | 1.50E-05 | 1.00E-05 | 5.00E-06 |
| 39 | Trametinib | 5.00E-02 | 4.00E-02 | 3.00E-02 | 2.00E-02 | 1.00E-02 | 1.00E-04 |
| 40 | Vandetanib | 5.00E-02 | 2.00E-02 | 1.00E-02 | 3.00E-03 | 1.00E-03 | 1.00E-04 |
| 41 | Vemurafenib | 1.00E-01 | 5.00E-02 | 2.00E-02 | 1.00E-02 | 3.00E-03 | 1.00E-03 |
| 42 | Vinblastine | 6.00E-06 | 5.00E-06 | 4.00E-06 | 3.00E-06 | 2.00E-06 | 1.50E-06 |
| 43 | Vincristine sulfate | 4.00E-05 | 6.00E-06 | 2.00E-06 | 1.50E-06 | 1.00E-06 | 5.00E-07 |
| 44 | Vinorelbine | 7.50E-05 | 5.00E-05 | 4.00E-05 | 3.00E-05 | 2.50E-05 | 2.00E-05 |

**Table B. SNPs significantly associated with drug response from MAGWAS after controlling for SNP rs1800566.**

SNPs associated with the multivariate response for each drug at the genome-wide suggestive significance level or higher from the genome-wide association using MAGWAS when controlling for the effects of SNP rs1800566 in the *NQO1* gene. The results are sorted by the host gene and drug. The SNPs that were not suggestively significant in the original genome-wide association mapping reported in Table 2 are shown in **bold**. Chr: Chromosome, The most severe consequences were obtained from Ensembl VEP (Ensembl release 97 – July 2019).

|  | **Drug** | **Chr** | **RSID** | **-log_10_**  **(pvalue)** | **Most severe consequence** | **Host gene symbol** | **Host gene Ensembl ID** |
| --- | --- | --- | --- | --- | --- | --- | --- |
| 1 | Cladaribine | 7 | rs540157 | 6.45 | regulatory_region_variant | *-* | - |
| 2 | Cladaribine | 9 | rs72706422 | 6.11 | intergenic_variant | *-* | - |
| 3 | Epirubicin | 10 | rs1125411 | 6.92 | intergenic_variant | *-* | - |
| 4 | Epirubicin | 10 | rs7911302 | 6.84 | intergenic_variant | *-* | - |
| 5 | Gemcitabine | 12 | rs11043377 | 6.24 | intergenic_variant | *-* | - |
| 6 | Gemcitabine | 12 | rs6486806 | 7.44 | intergenic_variant | *-* | - |
| 7 | Oxaliplatin | 10 | rs10826348 | 8.03 | intergenic_variant | *-* | - |
| 8 | Oxaliplatin | 10 | rs1112962 | 6.47 | intergenic_variant | *-* | - |
| 9 | Paclitaxel | 2 | rs1107718 | 7.49 | intergenic_variant | *-* | - |
| 10 | **Sorafenib** | **4** | **rs16883947** | **6.01** | **intergenic_variant** | ***-*** | **-** |
| 11 | Tivantinib | 18 | rs11662580 | 6.47 | intergenic_variant | *-* | - |
| 12 | Vandetanib | 7 | rs10273337 | 6.08 | intron_variant | *AGAP3* | ENSG00000133612 |
| 13 | **Etoposide** | **20** | **rs414644** | **6.01** | **non_coding_transcript_exon_variant** | ***AL121888.1*** | **ENSG00000286063** |
| 14 | **Vandetanib** | **14** | **rs11624985** | **6.05** | **non_coding_transcript_exon_variant** | ***AL358334.2*** | **ENSG00000258711** |
| 15 | Vemurafenib | 2 | rs4664521 | 6.50 | intron_variant | *CACNB4* | ENSG00000182389 |
| 16 | Vemurafenib | 2 | rs9784082 | 6.91 | intron_variant | *CACNB4* | ENSG00000182389 |
| 17 | **Paclitaxel** | **14** | **rs56794877** | **6.12** | **intron_variant** | ***CDKL1*** | **ENSG00000100490** |
| 18 | **Paclitaxel** | **3** | **rs7640846** | **6.04** | **intron_variant** | ***CNTN4*** | **ENSG00000144619** |
| 19 | Gemcitabine | 7 | rs216706 | 6.48 | intron_variant | *CREB5* | ENSG00000146592 |
| 20 | **Hydroxyurea** | **12** | **rs12810366** | **6.02** | **intron_variant** | ***GOLGA3*** | **ENSG00000090615** |
| 21 | Gemcitabine | 10 | rs17142881 | 6.68 | intron_variant | *ITIH5* | ENSG00000123243 |
| 22 | Vinblastine | 16 | rs1693956 | 6.24 | intron_variant | *LINC01081* | ENSG00000268754 |
| 23 | Dovitinib | 11 | rs7480726 | 6.02 | upstream_gene_variant | *MIR4299* | ENSG00000266645 |
| 24 | Dovitinib | 11 | rs7930221 | 6.75 | upstream_gene_variant | *MIR4299* | ENSG00000266645 |
| 25 | Oxaliplatin | 10 | rs11006706 | 9.12 | intron_variant | *MKX-AS1* | ENSG00000230500 |
| 26 | Carboplatin | 20 | rs6010746 | 6.22 | intron_variant | *MRGBP* | ENSG00000101189 |
| 27 | Erlotinib | 8 | rs2444306 | 6.19 | intron_variant | *OXR1* | ENSG00000164830 |
| 28 | **Temozolomide** | **2** | **rs2621472** | **6.09** | **intron_variant** | ***PLEKHM3*** | **ENSG00000178385** |
| 29 | Paclitaxel | 9 | rs4740816 | 6.15 | upstream_gene_variant | *PLGRKT* | ENSG00000107020 |
| 30 | Carboplatin | **21** | **rs11701367** | **6.01** | **intron_variant** | ***RUNX1*** | **ENSG00000159216** |
| 31 | Temozolomide | 3 | rs4854617 | 6.03 | intron_variant | *RYK* | ENSG00000163785 |
| 32 | Fluoro-deoxyuridine | 4 | rs10516497 | 6.59 | intron_variant | *SLC9B1* | ENSG00000164037 |
| 33 | Fluoro-deoxyuridine | 4 | rs9994654 | 7.17 | intron_variant | *SLC9B1* | ENSG00000164037 |
| 34 | Docetaxel | 5 | rs2304035 | 6.18 | missense_variant | *SLIT3* | ENSG00000184347 |

**Table C. Significant results from multivariate linear regression of drug response on the baseline expression of *NQO1* and *NFAT5* transcripts.**

Significant correlations between drug response and baseline expression of *NQO1* and *NFAT5* transcripts after multiple testing correction with a false discovery rate of q<0.25 applied per drug.

| **Ensembl Transcript Name** | **Ensembl Transcript ID** | **Drug** | **p-value** | **FDR corrected p-value** |
| --- | --- | --- | --- | --- |
| NQO1-206 | ENST00000564043 | Etoposide | 0.0122 | 0.0857 |
| NQO1-206 | ENST00000564043 | Arsenic trioxide | 0.0124 | 0.0869 |
| NQO1-206 | ENST00000564043 | Teniposide | 0.0190 | 0.1113 |
| NQO1-204 | ENST00000439109 | Ibrutinib | 0.0530 | 0.1247 |
| NQO1-204 | ENST00000439109 | Doxorubicin | 0.0217 | 0.1517 |
| NQO1-204 | ENST00000439109 | Docetaxel | 0.0903 | 0.2106 |
| NQO1-203 | ENST00000379047 | Crizotinib | 0.0008 | 0.0057 |
| NQO1-203 | ENST00000379047 | Ibrutinib | 0.0534 | 0.1247 |
| NQO1-203 | ENST00000379047 | Teniposide | 0.0733 | 0.1283 |
| NQO1-203 | ENST00000379047 | Vandetanib | 0.0250 | 0.1752 |
| NQO1-203 | ENST00000379047 | Docetaxel | 0.0617 | 0.2106 |
| NQO1-202 | ENST00000379046 | Teniposide | 0.0691 | 0.1283 |
| NQO1-201 | ENST00000320623 | Vinorelbine | 0.0080 | 0.0561 |
| NQO1-201 | ENST00000320623 | Teniposide | 0.0318 | 0.1113 |
| NQO1-201 | ENST00000320623 | MitomycinC | 0.0250 | 0.1747 |
| NQO1-201 | ENST00000320623 | Mitoxantrone | 0.0318 | 0.2225 |
| NQO1-201 | ENST00000320623 | Oxaliplatin | 0.0648 | 0.2267 |
| NFAT5-210 | ENST00000567990 | Ibrutinib | 0.0085 | 0.0594 |
| NFAT5-210 | ENST00000567990 | Docetaxel | 0.0812 | 0.2106 |
| NFAT5-202 | ENST00000354436 | Oxaliplatin | 0.0575 | 0.2267 |

**Table D. p-values for rs1800566 at each stage of the pQTL model.**

The p-values for the association of rs1800566 with the AUC for each drug treatment are shown in the Stage 1 column. The p-value for the association of rs1800566 with *NQO1* protein activity is shown in the Stage 2 column. The p-values for the association of *NQO1* protein activity with the AUC for each drug treatment are shown in the Stage 3 column. ‘*’ indicates statistical significance at p-value<0.05.

| **Drug treatment** | **p-values** | | |
| --- | --- | --- | --- |
|  | **Stage 1** | **Stage 2** | **Stage 3** |
| Arsenic trioxide | 0.015 * | 5.27e-10 * | 0.044 * |
| Erlotinib | 0.028 * |  | 0.157 |
| Paclitaxel + Epirubicin | 0.172 |  | 0.164 |
| Trametinib | 0.295 |  | 0.561 |

**Table E. Estimates and p-values for the covariate ‘NQO1_protein_activity’ from Stage 3 of the pQTL model for the linear regression of AUC on the *NQO1* protein activity stratified by genotype at SNP rs1800566.**

CC = reference genotype, CT = heterozygous genotype, TT = homozygous alternate genotype

| **Drug treatment** | **Genotype = CC** | | **Genotype = CT** | | **Genotype = TT** | |
| --- | --- | --- | --- | --- | --- | --- |
|  | **Estimate** | **p-value** | **Estimate** | **p-value** | **Estimate** | **p-value** |
| Arsenic trioxide | 0.493 | 0.349 | 0.537 | 0.877 | -2.838 | 0.514 |
| Erlotinib | 0.426 | 0.501 | 1.835 | 0.738 | -13.662 | 0.012 |
| Paclitaxel + Epirubicin | 0.135 | 0.724 | 0.345 | 0.890 | -8.346 | 0.063 |
| Trametinib | 0.142 | 0.733 | 1.504 | 0.582 | -6.254 | 0.148 |

**References**

1. Köhler U, Olbricht SS, Fuechsel G, Kettner E, Richter B, Ridwelski K. Weekly paclitaxel with epirubicin as second-line therapy of metastatic breast cancer: results of a clinical phase II study. Semin Oncol. 1997 Oct;24(5 Suppl 17):S17-40-S17-43.

2. Yang Y, Zhang Y, Wu Q, Cui X, Lin Z, Liu S, et al. Clinical implications of high NQO1 expression in breast cancers. J Exp Clin Canc Res. 2014 Dec;33(1):1-9.

3. Lin L, Qin Y, Jin T, Liu S, Zhang S, Shen X, et al. Significance of NQO1 overexpression for prognostic evaluation of gastric adenocarcinoma. Exp Mol Pathol. 2014 Apr 1;96(2):200-5.

4. Go WY, Liu X, Roti MA, Liu F, Ho SN. NFAT5/TonEBP mutant mice define osmotic stress as a critical feature of the lymphoid microenvironment. Proc Natl Acad Sci Unit States Am. 2004 Jul 20;101(29):10673-8.

5. Awadallah NS, Dehn D, Shah RJ, Russell Nash S, Chen YK, Ross D, et al. NQO1 expression in pancreatic cancer and its potential use as a biomarker. Appl Immunohisto M M. 2008 Jan;16(1):24-31.
